# Barriers and facilitators to the diagnosis of HIV and other STIs in primary care within publicly funded healthcare systems: A systematic review of qualitative studies

**DOI:** 10.1371/journal.pone.0341919

**Published:** 2026-02-05

**Authors:** Pilar Galicia, Maria Jesús García de Yébenes, Consuelo Pascau, Juan Cuadros González, Loreto Carmona, Jose-Manuel Ramos-Rincón

**Affiliations:** 1 Centro de Salud Segovia, Madrid, Spain; 2 Instituto de Salud Musculoesquelética (Inmusc), Madrid, Spain; 3 Freelance biomedical translator, proofreader and scientific writer, Madrid, Spain; 4 Servicio Microbiología Clínica, Hospital Universitario Alcalá de Henares, Madrid, Spain; 5 Facultad de Medicina Clínica, Universidad Miguel Hernández, Elche, Spain; Malaria Consortium, MOZAMBIQUE

## Abstract

**Background:**

Human immunodeficiency virus (HIV) and other sexually transmitted infections (STI) are a major public health concern. Primary care (PC) is the ideal setting for their diagnosis. Designing effective strategies requires a thorough understanding of the problems these patients face when seeking care.

**Objectives:**

To identify barriers and facilitators to the diagnosis of HIV and STIs in PC and classify them according to the theoretical Capability, Opportunity, and Motivation model of Behaviour (COM-B model). The review’s findings will inform the development of specific intervention strategies.

**Methods:**

We conducted a systematic review (SR) of qualitative studies of barriers and facilitators to the diagnosis of HIV/STI. A systematic literature search, without publication date limitation, was performed in Embase, Cochrane Library, and Medline via PubMed. The Joanna Briggs Institute (JBI) scale was used to assess the risk of bias of the included studies. The results were synthesised and presented according to the dimensions of the COM-B model.

**Results:**

Of the 1,366 records from the initial search strategy, 70 articles met all inclusion criteria, covering all HIV/STIs and different risk groups. Barriers and facilitators to diagnosis were identified at three levels: patients, professionals, and health system. Patient barriers included confidentiality issues, lack of risk perception, shame and stigma, and access. The barriers at the level of professionals were lack of training, time constraints, and scarcity of resources. Among the facilitators, the most notable were minimally invasive sampling methods, ensuring confidentiality, standardisation of tests, fast and efficient entry points, and professional training.

**Discussion:**

This SR provides information on barriers and facilitators to HIV/STI diagnosis in PC and across the different stakeholders involved (patients, providers, system). The results are grouped into the different dimensions of a behavioural model (capability, opportunity, and motivation).

A thorough understanding of the complexity of the behaviour of people diagnosed with HIV/STIs and all those involved in their management will enable the development of effective intervention strategies to diagnose and prevent these processes, thereby reducing the associated disease burden.

**Registration:** International Prospective Register of Systematic Reviews (PROSPERO: CRD42023404578).

## Introduction

In recent years, the number of sexually transmitted infections (STIs) has increased exponentially. According to the World Health Organisation (WHO), more than 374 million new infections were diagnosed in 2020 [1] and nearly 1 million people are infected daily with a curable STI [2]. In 2022, the increase in the number of reported STI cases over the previous 5 years in the United States (US) alone was 11% for gonorrhoea, 80% for syphilis, and 183% for congenital syphilis [3]. Young people aged 15–24 years account for almost 50% of newly reported STIs, and young women in particular account for 76% of chlamydia infections and 58% of gonorrhoea infections [4,5].

This increase in the incidence of STIs is a major public health challenge and a serious threat to high-risk patient populations. Although STIs are often asymptomatic, they can have adverse health effects, including infertility, pelvic inflammatory disease, chronic pelvic pain, and increased cancer risk. In addition, some STIs can affect pregnancy, leading to an increased risk of adverse outcomes [3]. A critical component of STIs control is testing. Screening for these infections in both symptomatic and asymptomatic populations is essential to reduce the associated morbidity, ensure adequate testing of contacts, and minimise the risk of human immunodeficiency virus (HIV) infection. The US Centers for Disease Control and Prevention and other institutions have issued recommendations for STI screening in the general population and in certain high-risk subgroups, and recommend opportunistic screening in general medical practice [6,7]. In this regard, the WHO global research agenda on STIs emphasized the need to adapt global STI research priorities to regional and setting-specific contexts [8].

In addition, molecular biology techniques have made it possible to obtain microbiological results within hours and even test for resistance in bacterial STIs. This allows for the use of less invasive techniques, such as urine screening or self-collection of vaginal exudates, as well as having key point-of-care (PoC) sites for diagnosis [9]. However, in a previous survey conducted by our group, a very low rate of sexual interviews and perception of STI frequency in the consultation was observed [10].

An ideal setting for such screening is primary care (PC) due to its accessibility, longitudinality, and transversality, but numerous barriers, such as beliefs, risk perception, and stigma, hinder early diagnosis and treatment of STIs at the first level of care [11,12]. For example, the geographical dispersion or delay in early detection of patients with STIs or asymptomatic STIs hinders the application of preventive measures, early diagnosis, and early treatment of contacts [13,14]. In addition, many clinicians fail not only to offer STI screening to young patients, but also to discuss sexual and reproductive health issues, such as STIs, HIV, or birth control [5].

The Capability, Opportunity, and Motivation model of Behaviour (COM-B model) is a theoretical framework for analysing behaviour visualised as a ‘behaviour change wheel’. This model breaks down behaviour into three components: *Capability* (physical and psychological), *Opportunity* (physical and social), and *Motivation* (reflective and automatic), which interact with each other. The application of this model to the STI diagnosis in PC allows us to understand the factors that influence whether individuals seek diagnosis and adhere to treatment. The *physical capability* expresses the need of patients to be physically able to attend clinical appointments, provide samples for testing, and adhere to treatment regimens, and the need of healthcare providers to perform examinations, collect samples, and administer treatments. The *psychological capability* refers to the need of patients to have the knowledge and understanding about STIs, their symptoms, and the importance of diagnosis and treatment, and the need of providers to have the knowledge and skills to accurately diagnose STIs, provide appropriate treatment, and communicate effectively with patients. *Physical opportunity* refers to the environmental and contextual factors that make the behavior possible such as access to healthcare services, availability of appointments, and affordability of tests and treatments for patients, and having the necessary resources and time to discuss STI diagnosis with patients for healthcare providers. *Social opportunity* involves the cultural and social influences that support or hinder the behavior including social norms around sexual health, stigma, and support from peers or family for patients, and support and guidelines provided by their healthcare system for providers. *Reflective Motivation* reflects conscious decision-making processes and evaluations that influence behavior involving the beliefs about the importance of STI diagnosis, the intentions to seek care, and the goals related to sexual health for patients, and the professional commitment to provide quality care and the intentions to follow best practices for STI diagnosis for providers. Lastly, *automatic motivation* refers to the emotional processes that influence behavior, such as feelings of embarrassment or fear related to STI diagnosis and habits related to seeking healthcare for patients, and emotional responses to discuss sexual health with patients for professionals. This framework is useful for identifying the factors that modulate behaviour and the interventions and implementation strategies that could be useful [15,16].

This behavioural model has already been applied to STIs. In 2018, McDonagh *et al.* published a systematic review (SR) of barriers and facilitators to chlamydia testing using the COM-B model. Barriers and facilitators were identified at the patient, provider, and service level and grouped into the dimensions of the model. However, this SR was limited to chlamydia and did not include studies on other STIs [17]. Although STIs are likely to have similar barriers and facilitators to their screening and diagnosis, it is important to analyse the different pathologies and risk groups to determine whether there are any distinctive elements.

To change daily clinical practice, it is necessary to integrate and evaluate the behaviour of the agents that influence change. Therefore, the use of the COM-B model can allow the development of interventions adapted to change behaviour, either at the level of the patient, the physician, or the health system.

The research question for our study was: What are the barriers and facilitators to HIV/STIs screening and diagnosis in PC? The primary objectives of this SR were to (1) identify the barriers and facilitators to HIV/STIs screening and diagnosis across the key stakeholders (patients, healthcare providers, and healthcare system) and (2) organize these factors within the framework of the COM-B model [16].

## Methods

### Protocol

This review was conducted according to PRISMA guidelines [18] (see S1 File (PRISMA checklist)) and the protocol was registered in the International Prospective Register of Systematic Reviews (PROSPERO: CRD42023404578).

### Inclusion and exclusion criteria

The question under review was defined according to the PICO (Patient, Intervention, Comparison, Outcome) system. STIs included those that can be routinely requested in primary care (HIV, syphilis, *Neisseria gonorrhoeae, Chlamydia trachomatis, Mycoplasma genitalium*). The inclusion criteria were as follows: *population*: adult patients with a diagnosis of HIV/STI or health care providers; *outcome*: evaluation of barriers or facilitators to diagnosis in PC; *study type*: qualitative studies conducted in countries where the model of health care delivery in PC is comparable to that in Spain to ensure that the results are relevant and applicable to the context in which the intervention will be developed.

The development of intervention strategies to increase STI diagnosis must account for local healthcare system characteristics, as differences in accessibility, funding, and equity influence intervention implementation and effectiveness. The Spanish healthcare system is public and universal, ensuring the entire population’s access to medical services without direct costs at the point of use. In Spain, general practitioner (GP) acts as a gatekeeper to access specialist services and general practice services are publicly funded.

This geographic and health-system selection criteria has been used by McDonagh et al in a SR on barriers to CT screening [19] and is supported by the the WHO recommendation to adapt STI research to regional and setting-specific contexts [8].

The exclusion criteria were primarily derived from the main purpose of the SR, *i.e.,* to identify the barriers and facilitators for all stakeholders involved in the diagnosis of HIV/STIs to design effective intervention strategies for PC in Spain. Local health system organization and incentives, such as enhanced services or dedicated staff, can impact uptake and sustainability of HIV/STI diagnostic interventions in PC [20]. Therefore, to increase the applicability of the results of the SR to the Spanish context, the following exclusion criteria were defined: a) type of population (children and pregnant women who were excluded since STIs screening is systematically performed during pregnancy in our health system); b) measurement of outcomes not related to the objectives (treatment or prognosis); c) non-qualitative design (clinical trials, advertising campaigns); d) studies conducted in countries with health systems not comparable to the country in which the intervention strategies are to be developed (Spain). For example, studies conducted in countries with private healthcare systems, such as the United States—where universal coverage is not guaranteed because healthcare is predominantly provided through employment-linked insurance—were excluded. African countries were also excluded, as their public healthcare systems are underfunded and also exists a predominance of traditional medicine, rendering them incomparable to European public systems, particularly the Spanish one.

### Search strategy and selection process

**Fig 1:**
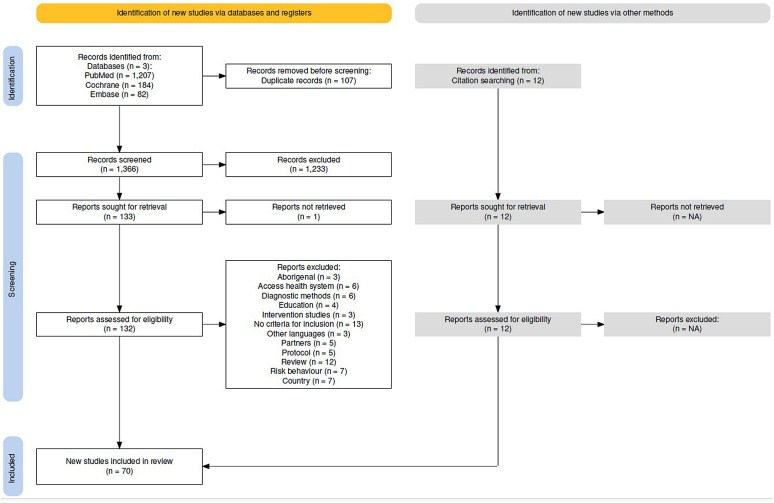
PRISMA 2020 flowchart of included studies.

The Embase, Medline via PubMed, and Cochrane Library databases were searched using free terms and controlled language up to December 1, 2023 (search strategy is available in S2 File). The process was peer review with subsequent discussion Fig 1 illustrates the selection process.

### Screening of studies, data collection, and synthesis

The retrieved references were managed in Raayan.ai© [21] to facilitate screening and consensus selection. Two reviewers (PG and CP) screened the studies and performed data collection and analysis. First, both reviewers independently reviewed all titles and abstracts against the selection criteria in 20-minute sessions. A third reviewer (MJGY) resolved disagreements between the reviewers. The full texts of the selected articles were then read in detail and subjected to a second selection process based on the *a priori* criteria.

The same reviewers collected data from the included studies in Excel files. Two types of data were extracted: 1) descriptive characteristics of the included articles (author, year and country, number of participants, type of STI, setting and sampling, sexual risk factors, population characteristics, theoretical model, method and type of analysis); 2) barriers and facilitators for patients, providers, and system categorised according to the COM-B model dimensions (capability, opportunity, and motivation).

### Quality assessment

The included articles underwent a quality assessment process using the Joanna Briggs Institute (JBI) critical appraisal tool for qualitative research [22]. The JBI scale consists of 10 items (Table 2) and was completed by the two independent reviewers, who reached consensus in case of disagreement.

**Table 2:** Quality of included studies assessed using the Joanna Briggs Institute critical appraisal tool.

AUTHOR, year	Q1	Q2	Q3	Q4	Q5	Q6	Q7	Q8	Q9	Q10
**Adams_2020 [56]**	Unclear	Yes	Yes	Unclear	Unclear	Yes	Unclear	Yes	Yes	Yes
**Adedimeji_2015 [57]**	Yes	Yes	Yes	Yes	Yes	Yes	No	Yes	Yes	Yes
**Ahmaro_2021 [23]**	Unclear	Yes	Yes	Yes	Yes	Yes	Unclear	Yes	Yes	Yes
**Ahmaro_2022 [78]**	Yes	Yes	Yes	Yes	Yes	Yes	Unclear	Yes	Yes	Yes
**Aicken_2016 [24]**	Yes	Yes	Yes	Unclear	Yes	Yes	Unclear	Yes	Yes	Yes
**Åkerman_2017 [58]**	Yes	Yes	Yes	Unclear	Yes	Yes	Unclear	Yes	Yes	Yes
**Allison_2017 [67]**	Yes	Yes	Yes	Yes	Yes	Yes	Unclear	Yes	Not needed	Yes
**Apers_2020 [68]**	Yes	Yes	Yes	Yes	Yes	Yes	Unclear	Yes	Yes	Yes
**Balfe_2009 [25]**	Yes	Yes	Yes	Yes	Yes	Yes	Unclear	Yes	Yes	Yes
**Balfe_2010 [26]**	Unclear	Unclear	Unclear	Yes	Unclear	Yes	Unclear	Yes	Yes	Yes
**Bedert_2021 [27]**	Yes	Yes	Yes	Yes	Yes	Yes	Unclear	Yes	Yes	Yes
**Bilardi 2013 [47]**	Yes	Yes	Yes	Yes	Yes	Yes	Unclear	Yes	Yes	Yes
**Blondell_2021 [59]**	Yes	Yes	Yes	Yes	Yes	Yes	No	Yes	Yes	Yes
**Boyce_2012 [48]**	Yes	Yes	Yes	Yes	Yes	Yes	Unclear	Yes	Yes	Yes
**Boydell_2017 [28]**	Yes	Yes	Yes	Yes	Yes	Unclear	Unclear	Yes	Yes	Yes
**Brendstrup_1990 [29]**	Yes	Unclear	Yes	Yes	Unclear	Unclear	Unclear	Yes	Not mentioned	Yes
**Brugha_2011 [82]**	Unclear	Unclear	Unclear	Unclear	Unclear	Yes	Unclear	Yes	Yes	Yes
**Christianson_2010 [30]**	Yes	Yes	Yes	Yes	Yes	Yes	Unclear	Yes	Yes	Yes
**Day_2011 [79]**	Yes	Unclear	Yes	Unclear	Yes	Yes	No	Yes	Yes	Yes
**Denison_2017 [31]**	Yes	Yes	Yes	Yes	Yes	Yes	No	Yes	Yes	Yes
**Dowson_2012 [49]**	Yes	Yes	Yes	Yes	Yes	Unclear	Unclear	Yes	Not mentioned	Yes
**Etowa_2022 [60]**	Yes	Yes	Yes	Yes	Yes	Yes	Unclear	Yes	Yes	Yes
**Evans_2016 [61]**	Yes	Yes	Yes	Yes	Yes	Yes	No	Yes	Yes	Yes
**Ewert_2016 [32]**	Unclear	Yes	Unclear	Unclear	Yes	Yes	Unclear	Yes	Yes	Yes
**Fdez•Gerlinger_2013 [92]**	Yes	Yes	Yes	Yes	Yes	Yes	Unclear	Yes	Yes	Yes
**Figueira_2022 [83]**	Yes	Yes	Yes	Yes	Yes	Yes	Unclear	Yes	Yes	Yes
**Fleming_2020 [33]**	Yes	Yes	Yes	Yes	Yes	Yes	No	Yes	Yes	Yes
**Flowers_2017 [50]**	Yes	Yes	Yes	Yes	Yes	Yes	No	Yes	Yes	Yes
**Godin_2000 [51]**	Yes	Yes	Yes	Yes	Yes	Yes	Unclear	Yes	Yes	Yes
**Grandahl_2020 [89]**	Yes	Yes	Yes	Yes	Yes	Yes	No	Yes	Yes	Yes
**Heijman_2017 [52]**	Yes	Yes	Yes	Yes	Yes	Yes	No	Yes	Yes	Yes
**Heritage_2008 [34]**	Unclear	Yes	Unclear	Unclear	Unclear	Unclear	Unclear	Yes	Yes	Yes
**Hocking_2008 [69]**	Yes	Yes	Yes	Yes	Yes	Yes	No	Yes	Yes	Yes
**Hogan_2010 [35]**	Yes	Yes	Yes	Yes	Yes	Yes	No	Yes	Yes	Yes
**Jones_2017 [36]**	Yes	Yes	Yes	Yes	Yes	Yes	No	Yes	Yes	Yes
**Joore_2017 [70]**	Yes	Yes	Yes	Yes	Yes	Yes	No	Yes	Yes	Yes
**King_2017 [66]**	Yes	Yes	Yes	Yes	Yes	Yes	Unclear	Yes	Yes	Yes
**Krabbenborg_2021 [84]**	Yes	Yes	Yes	Yes	Yes	Yes	No	Yes	Yes	Yes
**Lorch_2015_b [80]**	Yes	Yes	Yes	Yes	Yes	Yes	Unclear	Yes	Yes	Yes
**Lorimer_2009 [38]**	Yes	Yes	Yes	Yes	Yes	Yes	Unclear	Yes	Yes	Yes
**Lorimer_2013 [37]**	Yes	Yes	Yes	Yes	Yes	Yes	No	Yes	Yes	Yes
**Lorimer_2014 [71]**	Unclear	Yes	Unclear	Unclear	Yes	Yes	Unclear	Yes	Yes	Yes
**Malta_2007 [39]**	Yes	Yes	Yes	Yes	Yes	Yes	No	Yes	Yes	Yes
**Manirankunda_2009 [53]**	Yes	Yes	Yes	Yes	Yes	Yes	Unclear	Yes	Yes	Yes
**Manirankunda_2012 [72]**	Yes	Yes	Yes	Yes	Yes	Yes	Unclear	Yes	Yes	Yes
**Masaro_2012 [73]**	Yes	Yes	Yes	Yes	Yes	Yes	No	Yes	Yes	Yes
**McDonagh_2020 [15]**	Yes	Yes	Yes	Yes	Yes	Yes	No	Yes	Yes	Yes
**McNulty_2004 [74]**	Yes	Yes	Yes	Yes	Yes	Yes	No	Yes	Yes	Yes
**McNulty_2010 [75]**	Yes	Yes	Yes	Yes	Yes	Yes	No	Yes	Yes	Yes
**Mills_2006 [90]**	Unclear	Yes	Unclear	Unclear	Unclear	Yes	Unclear	Yes	Yes	Yes
**Mitra 2006 [62]**	Yes	Yes	Yes	Yes	Yes	Yes	No	Yes	Yes	Yes
**Navaza_2012 [54]**	Unclear	Yes	Unclear	Yes	Yes	Yes	No	Yes	Yes	Yes
**Normansell_2016 [40]**	Yes	Yes	Yes	Yes	Yes	Yes	No	Yes	Yes	Yes
**Oliver de Visser_2013 [41]**	Yes	Yes	Yes	Yes	Yes	No	Unclear	Yes	Yes	Yes
**Peters_2022 [55]**	Yes	Yes	Yes	Yes	Yes	Yes	No	Yes	Yes	Yes
**Prost_2007_a [85]**	Yes	Yes	Yes	Yes	Yes	Yes	No	Yes	Yes	Yes
**Prost_2007_b [63]**	Yes	Yes	Yes	Yes	Yes	Yes	Unclear	Yes	Yes	Yes
**Rana_2022 [12]**	Yes	Yes	Yes	Yes	Yes	Yes	No	Yes	Yes	Yes
**Reisen_2014 [86]**	Yes	Yes	Yes	Yes	Yes	Yes	Unclear	Yes	Not mentioned	Yes
**Scheim_2017 [88]**	Yes	Yes	Yes	Yes	Yes	Yes	Unclear	Yes	Yes	Yes
**Seedat_2014 [64]**	Yes	Yes	Yes	Yes	Yes	Yes	No	Yes	Yes	Yes
**Shangase_2015 [65]**	Yes	Yes	Yes	Yes	Yes	Yes	No	Yes	Yes	Yes
**Shoveller_2009 [42]**	Yes	Yes	Yes	Yes	Yes	Yes	No	Yes	Yes	Yes
**Somerset_2021 [43]**	Yes	Yes	Yes	Yes	Yes	Yes	No	Yes	Yes	Yes
**Thornton_2012 [81]**	Yes	Yes	Yes	Yes	Yes	Yes	Unclear	Yes	Yes	Yes
**Uusküla_2006 [44]**	Yes	Yes	Yes	Yes	Yes	Yes	Unclear	Yes	Yes	Yes
**Vaughan_2010 [45]**	Yes	Yes	Yes	Yes	Yes	Yes	No	Yes	Yes	Yes
**Wagg_2020 [46]**	Yes	Yes	Yes	Yes	Yes	Yes	Unclear	Yes	Yes	Yes
**Wallace_2012 [76]**	Yes	Yes	Unclear	Yes	Unclear	Unclear	Unclear	Yes	Yes	Yes
**Woodbridge_2015 [77]**	Yes	Yes	Yes	Yes	Yes	Yes	Unclear	Yes	Yes	Yes

Q1: Is there congruity between the stated philosophical perspective and the research methodology?

Q2: Is there congruity between the research methodology and the research question or objectives?

Q3: Is there congruity between the research methodology and the methods used to collect data?

Q4: Is there congruity between the research methodology and the representation and analysis of data?

Q5: Is there congruity between the research methodology and the interpretation of results?

Q6: Is there a statement locating the researcher culturally or theoretically?

Q7: Is the influence of the researcher on the research, and vice versa, addressed?

Q8: Are participants, and their voices, adequately represented?

Q9: Is the research ethical according to current criteria or, for recent studies, is there evidence of ethical approval by an appropriate body?

Q10: Do the conclusions drawn in the research report flow from the analysis, or interpretation, of the data?

## Results

We identified 1,473 studies, of which 107 were duplicates. Of the remaining 1,366, 1,233 were excluded after reading the title and abstract. A total of 12 articles were retrieved by secondary search, mainly from previous SRs. After full-text reading, 70 studies were included (Fig 1). Reasons for exclusion are available in S2 File (Table S4) and S3 File (Complete search articles). The characteristics of the 70 included studies are described in Table 1, the quality assessment in Table 2, and the main results in Tables 3 and 4.

**Table 1:** Overview of the studies included in the systematic review.

#	Author, year	Country	N	STI	Setting Sampling	Sexual risk factors	Population	Theoretical model	Method	Analysis
1	Adams, 2020 [56]	New Zealand	19	HIV	Social media recruitment	•Gay •Bisexual	•Filipino men (migrants) •Well educated	Guidelines of Massey University	Face-to-face interview	Content Inductive
2	Adedimeji, 2015 [57]	Irland	60	HIV	Snowball and convenience		•African immigrants •Age: 18–64 years •Postgraduates: 1/3 •Employed: 2/3		Focus group Semi-structured interview	Thematic
3	Ahmaro, 2021 [23]	UK	26	CT	•4 youth centres •Purposive		•Young people’s opinion on CT testing in pharmacies •Men/women: 16/10 •Age: 16–24 years		Face-to-face interview	Thematic
4	Ahmaro, 2022 [78]	UK	22	CT	•Community pharmacies •Purposive		•Pharmacists •Men/women: 11/11 •Provider CT testing: 73%	NPT	Semi-structured interview	Thematic
5	Aicken, 2016 [24]	UK	25	STIs	•Community/GP •Purposive	•Heterosexual •Bisexual •Gay	•Men/women: 16/9 •Black (21), mixed (3), Asian (1) •Age: 16–23 years		In-depth interview	Thematic Inductive-deductive
6	Åkerman, 2017 [58]	Sweden	19	HIV	•Schools •Workplaces •Purposive		•Thai immigrant women (19) •Age: 24–50 years •Second school/university: 68%	Andersen’s behavioral model	In-depth interview	Thematic
7	Allison, 2017 [67]	UK	26	•3C •HIV	•GP staff		•GP (9), PN (13), manager (3), receptionist (1) •Men/women: 5/23 •Age: 30–60 years	Modified conceptual framework for fidelity of implementation	Semi-structured interview	Thematic Deductive
8	Apers, 2020 [68]	Belgium	122	HIV	•Regional GP •Convenience		•GP (52% women) •Median age: 51 years		Focus group	Thematic
9	Balfe, 2009 [25]	Ireland	30	STIs	•GP services •Purposive	MSM	•Men/women: 9/20 •Age: 18–21 years		Semi-structured interview	Thematic
10	Balfe, 2010 [26]	Ireland	35	CT	•GP and family planning clinics •Rural and urban		•Young women (35) •Age: 18–29 years		Semi-structured interview	Thematic
11	Bedert, 2021 [27]	The Netherlands	14	HIV	•HIV centres •Community	MSM	•Men/women: 11/3 •Median age: 46 years		In-depth interview	Thematic and content
12	Bilardi, 2013 [47]	Australia	31	HIV	•AIDS council organisation •Opportunistic	MSM	•MSM views on self-testing •Age: < 30 years/ > 30 years: 15/16 •Homosexual (28), bisexual (3)	Interpretative phenomenological analysis	Semi-structured interview	Thematic
13	Blondell, 2021 [59]	Australia	10	HIV	•Targeted location sampling		•Vietnamese migrants •Men/women: 4/6 •Age: 18–49 years	Phenomenological analysis	Semi-structured interview	Thematic
14	Boyce, 2012 [48]	Guatemala	29	STIs	•NGO sexual minorities •Purposive	MSM Sex worker	•MSM •Transgender (8), gay/bisexual (16), heterosexual (5) •Age (mean): 29 years •University education (34%)		Semi-structured interview	Thematic
15	Boydell, 2017 [28]	UK	30	HIV	•Community organizations •Snowballing	•MSM •Gay •Bisexual	•Men •Age: 18–29 years	Framework approach	Semi-structured interview	Thematic
16	Brendstrup, 1990 [29]	Denmark	10	HIV	•Media advertisement	•Homosexual •Bisexual	•Men Age: 26–46 years	Lazarus framework (coping with stressful situations)	In-depth interview	
17	Brugha, 2011 [82]	Ireland	6065	CT	•Community •University		•HSA (400), students (5685) •Age: 18–29 years •Female: HSA 76.5% •Students: 59.8%		Survey	Descriptive statistics
18	Christianson, 2010 [30]	Sweden	23	HIV	•Youth clinic	•Homosexual •Bisexual	•Young people •Men/women: 9/14 •Age: 18–24 years		Focus group	Content
19	Day, 2011 [79]	Australia		STIs	Nurses working with IDUs	•IDU •Unprotected sex	•Nurses working with IDUs		Semi-structured interview	
20	Denison, 2017 [31]	New Zealand	24	STIs	University clinic		•University students •Men/women: 7/16, queer (1) •Age: 19–32 years •Various ethnicities		Semi-structured interview	Thematic
21	Dowson, 2012 [49]	UK	17	HIV	Hospital HIV outpatients	•MSM	•MSM •CD4 < 200 or AIDS-defining disease •Age: 33–67 years		Semi-structured interview	Thematic
22	Etowa, 2022 [60]	Canada	107	•HIV •PrEP •PEP	Community		•ACB people •Community members •Leaders, providers •Decision-makers	Socio-ecological model Intersectionality theory	Focus group	Thematic
23	Evans, 2016 [61]	UK	48	HIV	•Community •Purposive		•African migrants •Men/women: 19/29 •Age: 18–45 years	Health belief model	Focus group	Thematic
24	Ewert, 2016 [32]	Australia	28	STIs	University	•Heterosexual •Same sex attracted	•Male students: 18–25 years •Australian (23), international (5)		Semi-structured interview	Content and Thematic
25	Fdez- Gerlinger, 2013 [92]	France	24	HIV	GP		•Patients seen by GP •Men/women: 11/13 •Age (mean): 41 years		Semi-structured interview	Thematic Inductive approach
26	Figueira, 2022 [83]	Portugal	210 15	HIV	•Community pharmacies •PoC in pharmacies		•PoC users (210): -men (64%), mean age: 35 ± 13 years •Pharmacists (15): -women (73%), mean age: 38 years		Survey (PoC users) Focus group (pharmacists)	Thematic
27	Fleming, 2020 [33]	UK	26 3	•CT •NG	•On-site screening •Purposive		•College students (26) - Women: 62% - Age: 17.5 ± 1.8 years - Ethnicity: Black 50% •Teaching staff (3)		Semi-structured interview	Thematic
28	Flowers, 2017 [50]	UK	55	HIV	•NHS offices •Voluntary organisations •University	MSM	•MSM (self-test) •Provider		Focus group	Thematic
29	Godin, 2000 [51]	Canada	20	HIV	•Community	Homosexual	•Gay men •Age:18–45 years	Planned behavior	Focus group	Thematic
30	Grandahl, 2020 [89]	Sweden	20	•CT •NG	•National eHealth website •Purposeful sample		•Individuals ordering self-sampling test •Men/women: 9/11 •Age:18–49 years	Health belief model	Semi-structured interview	Deductive approach
31	Heijman, 2017 [52]	The Netherlands	30	STIs	STI outpatient clinic	MSM	•MSM after HIV diagnosis •Age (median): 38 years •High education: 68%	•Information-motivation behavior •Integrating social context in health-behavior interventions	Semi-structured interview	Inductive approach
32	Heritage, 2008 [34]	UK	18	CT	•School, GP •Purposive		•Young people (age: 15–18 years) •School (12), GP (6) •Various ethnicities		Focus group Individual interview	Long-table approach
33	Hocking, 2008 [69]	Australia	21	CT	•National database •Random sample		•GP •Women (8)		Survey Semi-structured interview	Thematic
34	Hogan, 2010 [35]	UK	36	CT	•GP offices •Purposive		•Young people •Men/women: 9/27 Age: 15–24 years	Planned behavior	Semi-structured interview	Thematic
35	Jones, 2017 [36]	UK	30	•CT •HIV	•GP practices •Purposive		•Young people •Men/women: 9/21 •Age: 16–24 years	Planned behavior	Face-to-face interview	Thematic
36	Joore, 2017 [70]	The Netherlands	90	HIV	•GPs’ opinion about guidelines		•GP with interest in STIs/HIV (9) - men 44%, age (median): 46 years - HIV patients in practice >25 years: 44% •Other GP - men 52%, age (median) 51 years - HIV patients in practice >25 years: 6%		Semi-structured interview Focus group	Framework
37	King, 2017 [66]	Russia	29	HIV	•NGO •Purposive	Sex workers IDU	•Female sex workers •Age: 21–38 years •IDU: 99%	Health belief	In-depth interview	Thematic
38	Krabbenborg, 2021 [84]	The Netherlands	15	HIV	•Rapid tests (checkpoint) •Community •Convenience	MSM	•Lay providers (10) -age (mean): 35.0 years •Patients (5) -age (mean): 28.6 years		Semi-structured interview	Thematic Brand and Clark approach
39	Lorch, 2015 b [80]	Australia	23	CT	•PN (rural) •Purposive sampling		•PN: women (96%) •Age: 30–59 years •CT testing experience: 12 years		Semi-structured interview	Thematic
40	Lorimer, 2009 [38]	UK	24	CT	Non-medical setting		•Men/women: 19/14 •Opinion about urine screening •Age: 16–24 years		In-depth interview	Thematic
41	Lorimer, 2013 [37]	UK	60	CT	•Non-clinical setting •Purposive and snowball		•Heterosexual men •Views on internet screening •Age: 16–24 years •Deprived and non-deprived areas		Focus group	Thematic Framework
42	Lorimer, 2014 [71]	UK	18	CT	•GP •Purposive		•GP staff: views on internet screening •GP (10), PN (8)		Semi-structured interview	Thematic Framework analysis
43	Malta, 2007 [39]	Brazil	30	STIs	•Public clinics •Purposive	MSM	•Heterosexual women (10) -age (median): 32 years •Heterosexual men (10) -age (median): 21 years •MSM (10), age (median): 21 years •Low level of education		Semi-structured interview	Thematic Content
44	Manirankunda, 2009 [53]	Belgium	70	HIV	•Community •Purposive		•SAM •Men/women: 33/36 •Age (mean): 22 years •Health insurance: 83%		Focus group	Content analysis Inductive
45	Manirankunda, 2012 [72]	Belgium	20	HIV	•Community •Purposive		•Physicians (GPs and internists) •Opinion about SAM •Men/women: 12/8 •Age: 42 (GPs), 44 (internists)	Grounded theory	Semi-structured interview	Inductive
46	Masaro, 2012 [73]	Canada	21	STIs	•Youth clinic •STI clinic •Reproductive clinic •Public health units		•STI providers •Men/women: 3/18 •Age: 23–65 years •GPs (5); nurses (14) •Administrative (1)		Semi-structured interview	Ethnographic Thematic
47	McDonagh, 2020 [15]	UK	28	CT	•GP •Purposive and convenience		•Young people •Age: 16–24 years •Women (18) •Heterosexual (19)	Behavior change wheel	Semi-structured interview	Thematic Inductive
48	McNulty 2004 [74]	UK	12	CT	GP		•GP staff: GP, PN, midwives •Practice managers	Grounded theory	Focus group	Iterative approach
49	McNulty, 2010 [75]	UK		CT	•GP •Purposive		•GP staff: -GP (72), nurses (46) - receptionist (23) - practice manager (8) - others (7)	TPB	Focus group	Framework approach
50	Mills, 2006 [90]	UK	45	CT	Purposive		•Views on postal screening •Men/women: 19/26 •Age: 16–39 years	Grounded theory	In-depth interview	Thematic Inductive
51	Mitra, 2006 [62]	Canada	20	HIV	•Outpatient clinics •Medical facilities for HIV testing •Purposive		•VCT •Immigrant women from endemic countries (8) •VCT practitioners (12)	Ottawa decision support framework	Semi-structured interview	Thematic
52	Navaza, 2012 [54]	Spain	13	HIV	•Hospital and NGOs •Purposive		•SAM •Men/women: 12/1 •Age: 22–34 years •Low level of education •Muslim: 86%	Health action process	Focus group Semi-structured interview	Ethnographic
53	Normansell, 2016 [40]	UK	17	STIs	•Community (college) •Semi-purposive		•Women •Age: 16–27 years •Ethnicity: White 35%, Black 30%, mixed 24%	3 theories: candidacy, planned behavior, stigma	Semi-structured interview	Thematic
54	Oliver de Visser, 2013 [41]	UK	8	STIs	•Purposive		•Young people •Age: 17–25 years •Men/women: 3/5	Phenomenological analysis	Interview	Thematic
55	Peters, 2022 [55]	The Netherlands	20	STI	•STI clinic •Internet •Men saunas •Purposive	MSW-MSM	•MSW (20) •Identity men/women (18/2) •Age: 18–66 years	Health belief model Reasoned action approach	Semi-structured interview	Thematic
56	Prost, 2007_a [85]	UK	24	HIV	•Gay venues •Purposive	MSM	•Rapid HIV test in non-clinical setting -Gay venue owners (6), -Gay users (4), -Providers (1) -Rapid test to MSM -Gay men VIH+	Exploratory	Semi-structured interview Focus group	Thematic
57	Prost, 2007_b [63]	UK	42	HIV	•African communities •Purposive		•HIV-VCT •Africans in London - Age: 18–29 years -Young people (8) -Women (6), mixed gender (11) -Francophone (6) -VIH+ (11)	Framework approach	Focus group	Thematic
58	Rana, 2022 [12]	Canada	27	STIs	•Professional network of HIV/AIDS •Purposive	GBM	•Age: −18–30 years (40%) − 30–50 years (30%) •White (48%) •HIV+(59%)	Pragmatic paradigm	Semi-structured interview Focus group	Thematic
59	Reisen, 2014 [86]	Colombia	32	HIV	Targeted recruitment	MSM	•Key informants (12) •MSM (20) •Mean age: 26 years •At least high school		In-depth interview	Thematic
60	Scheim, 2017 [88]	Canada	40	•HIV •STIs	•Community •Convenient and quota sampling	MSM - heterosexual - homosexual - bisexual - queer	•Gender identity: male (68%), trans man (80%), queer (18%) •Age: 18–34 years (73%)		Semi-structured interview	Thematic
61	Seedat, 2014 [64]	UK	20	HIV	•Migrant community groups •Purposive		•Migrants •Mean age: 43 years •Men/women: 10/10 •Fluent in English: 5	Framework approach	Semi-structured interview	Thematic
62	Shangase, 2015 [65]	UK	30	HIV	•African community organizations •Purposive		•African migrants •Men/women: 12/18 •Age: 21–65 years •Good level of education	Kleinman’s model of health care systems	Focus group	Thematic
63	Shoveller, 2009 [42]	Canada	70 22	STIs	•Community •Purposive		•Young people (70) -men/women: 33/37 -age: 15–24 years •Health care providers (22): -White nurses-GP: 68%−23%		In-depth interview Naturalistic observations	Thematic
64	Somerset, 2021 [43]	UK	338	HIV	•Construction facilities •Convenience		•Construction workers - mostly men - age (mean): 40 years - White (83%) - heterosexual (97%)	Interpretive philosophical orientation	Semi-structured interview Focus group	Thematic
65	Thornton, 2012 [81]	UK	Pre-test: 45 Post-test: 44	HIV	4 healthcare settings: - emergency - acute admission - dermatology - primary care		•Staff attitudes to routine testing - doctors - nurses - administrative - laboratory		Focus group	Thematic
66	Uusküla, 2006 [44]	Estonia	29	STIs	•Outpatient STI services •Convenience		•Men/women: 13/16 •Age (median): 24 years •University/technical students •Men living in rural area		Focus group	Thematic
67	Vaughan, 2010 [45]	Ireland	7	CT	•Free on-campus screening •Mass recruitment (mail)		•University students •Men/women: 1/6 •Age: 18–29 years		Semi-structured interview	Thematic
68	Wagg, 2020 [46]	Australia	11	CT	•Regional and rural •Invitation leaflets		•Sexually active women •Age: 18–30 years •Students and professionals	Social constructionist	Semi-structured interview	Thematic
69	Wallace, 2012 [76]	UK	55	CT	•GP staff		•GP (18) -PN (26) -receptionists (9) -practice manager (1) -research nurse (1)	Planned behavior	TPB questionnaire Semi-structured interview	Thematic Quantitative
70	Woodbridge, 2015 [77]	New Zealand	17	STIs	•Testing for men •Purposive		•GPs’ perceptions		Semi-structured interview	Thematic

3C = Chlamydia screening, signposting to contraceptive services, free condoms; ACB = African, Caribbean, and Black; AIDS = Acquired immune deficiency syndrome; CD4 = Cluster of differentiation 4; CT = *Chlamydia trachomatis*; GBM = Sexually active gay, bisexual, or other men who have sex with men; GP = General practice/practitioner; HIV = Human immunodeficiency virus; HSA = Healthcare setting attendee; IDU = Injecting drug user; MSM = Men who have sex with men; MSW = Male sex worker; NG = *Neisseria gonorrhoeae*; NGO = Non•governmental organisation; NHS = National Health Service; NPT = Normalisation process theory; PEP = Post•exposure prophylaxis; PN = Practice nurse; PoC = Point•of•care; PrEP = Pre•exposure prophylaxis; SAM = Sub•Saharan African migrant; STI = Sexually transmitted infection; TPB = Theory of planned behavior; UK = United Kingdom; VCT = Voluntary counselling and testing

**Table 3:** Synthesis of results: barriers to the diagnosis of sexually transmitted infections for different stakeholders according to the Capability, Opportunity, and Motivation model of Behavior (COM•B model) dimensions.

	COM•B component	Patient	Provider	System
**CAPABILITY**	***Physical*** Skills, abilities, or proficiencies	•Genital examination [31] •Physical skills [15,50] •Surveillance of behavior [33]	•Expertise or competence [34,40,61,63,68,75] •Skills to collect sample for testing [83] •Type of test [50,69] •Visit for unrelated sexual problems [76]	•Care pathways (follow•up) [63]
	***Psychological*** Knowledge, memory, attention, decision processes, behavioral regulation	•Accuracy of tests [47,50] •Attitude [23,50,51,53,55,57,60,79,88] •Behavior [33,64,71,73,77,85,86] •Concerns [26,34,56,62], confidentiality [32,42,44,59,60,62,64,66,73,80,85,89] •Forgetfulness [35,79] •Information/knowledge [15,33,35,44–46,52,57,61,69,86,89] •Language [12,28,54,57,58,61,62,65,76,86,89]	•Attitude [51,60,64,68,74,75,79,80] •Concerns about patients’ feelings [68–70,72,75–77] •Confidentiality [42,73,81] •Communication [27,30,39,65,68,69,71,72,74,76,77] •Forgetfulness [67,76] •Information/knowledge [12,23,27,30,39,60,65,68–70,72–75,81,83,84,88] •Ethical issues [72]	•Bureaucracy [86] •Confidentiality [34,35,64,75] •HIV exceptionalism [70,72] •Information [32,40,46,53,56,62,65,83,86,92] •Language [57,62,72] •Ethical issues [72]
**MOTIVATION**	***Reflective*** Beliefs about capabilities and consequences, roles, identity, intentions, goals, optimism	•Beliefs [29,32,41–44,50,52,54,55,60,61,63–66,88,92] •Guilt [15,34,46,66] •Risk [15,25,27,31,33,38,41,43,46,47,49,53,55,58,59,64,66,92] •Testing not a priority [15,31,66]	•Risk [77,88]	•Low awareness [43,55,64]
	***Automatic*** Emotions, reinforcement such as rewards, incentives, punishment	•Anxiety [24,25,28,44,51,63,66,90] •Discomfort [12,42,45,52,85,88] •Embarrassment [15,24,25,31–33,35,36,38,40,41,44–46,55,56,69,79,86,90,92] •Employment [53,66] •Fear [12,15,27,29,31,35,39,40,48,49,51–55,60–64,66,70,79,86,88,92]	•Lost opportunities for engagement in the system [50] •Need of follow•up [73,86] or contact tracing [74] •Frustration [73]	
**OPPORTUNITY**	***Physical*** Environmental context and resources	•Access (7, 19, 31, 33, 34, 47–49, 52, 53, 74, 79) •Financial [43,46,47,53,57,64–66,86] •Geographical [12,46,54,57,66] •Time constraints [12,36,40,42–45,47,48,57,60,66,79,86]	•Specific clinics [75] •IC testing not applicable [70] •Time constraints [15,34,35,44,62,67,68,70,72–77,80,81]	•Access [12,15,27,30,31,40,61,83] •Financial [25,31,32,53,57,62,64,66,69,70,74,82,86,92] •Resources [15,27,39,42,56,67,68,73–75,88] •Time constraints [12,15,44,61,68,69,72–74]
	***Social*** Social influence, pressure, norms, conformity, comparisons	•Concerns [24,34,45,62,85] •Culture [36,53,54,61,62,64,65,69,72,92] •Disclosure [15,26,40,42,51,55,79,83,88] •Discrimination/stigma [12,15,25,28,31,38,39,41,42,49,51,54–57,60–62,64,65,70,83,85,86,90] •Gender [12,42,48,65,71,88] •Judgement from professionals [25,27,40,46,70,79] •Lack of support [43,47,48,57,62,64,69,86] •Peers [23,33] and social norms [28,55] •Poor provider•patient relationship [51,57,62,74,93]	•Culture [40,60,62,64,72,77] •Discrimination/stigma [12,42,56,57,60,64,81] •Family members [75,76]/ disclosure of risk behaviour [77] •Judgement from professionals [46,60,70] •Presence of family [75,76] and disclosure [77] •Poor continuity of care [62]	•Discrimination/stigma [12,57,64,81] •Gender issues [12,42,88] •Lack of privacy in the reception area [34,67] •Lack of support/trust [57,61,64,69,75] •Screening by non•medical professionals [26,75,78,82]

HIV=Human immunodeficiency virus; IC=Indicator condition

**Table 4:** Synthesis of results: facilitators to the diagnosis of sexually transmitted infections for different stakeholders according to the Capability, Opportunity, and Motivation model of Behavior (COM•B model) dimensions.

	COM•B component	Patient	Provider	System
**CAPABILITY**	***Physical*** Skills, abilities or proficiencies	•Never been tested [83]	•Technical skills [74,83] •Young professionals [34]	•Training [15,62,63,67–69,72,74,75,77,80,81,83,84]
	***Psychological*** Knowledge, memory, attention, decision processes, behavioral regulation	•Awareness (25, 28, 34, 45, 54). •Confidentiality (7, 28, 31, 50, 53, 74, 80) •Information (6, 9, 14, 18, 34, 45) •Symptoms (15, 29, 49) •Normalisation of testing [40,41,87,88]	•Characteristics of providers [15,23,31,32,35,36,40,59,69,72,80,83,84] •Information [12,15,39,40,55,60,70,75,81] •Normalisation of testing [12,26,70,81]	•Methods [15,34,45,55,71,74,82,85] •Confidentiality [24,37,45,64,71,83] •Education [15,25,32,33,40,44,49,60–63,69,84,89] •Information [12,15,44,55,58,60,61,64,74,75,85,89]
**MOTIVATION**	***Reflective motivation*** Beliefs about capabilities and consequences, roles, identity, intentions, goals, optimism	•Risk [12,25,33,43,46,55,59,86] •Possibility of getting treatment [61,66] •Responsibility [25,28,55,66,74,86,88] •Trust [29,36,44,55,61,84] •Reduction of stress and anxiety [43,45,47,51,66,90]	•Responsibility [15] •Trust [61,72,84]	
	***Automatic motivation*** Emotions, reinforcement such as rewards, incentives, punishment	•Vulnerability to STIs [12,25,28,52,83,88] •Employment [25,66] •Emotional preparation [79]	•Incentive payment [69] •Positive reinforcement [15] •Empowerment of patients [80]	•Incentives [33]
**OPPORTUNITY**	***Physical opportunity*** Environmental context and resources	•Access [40,42,70,79,83,87,88] •Convenience [23,38,40,43,45,59,71,84,89] •Financial [40,67,89,94] •Time [12,40,43,47,63,83,84,86,89]	•Convenience [44,62,76] •Referral [63,79]	•Access [12,23,24,30,33,37,41,45,49,50,62–64,75,79,82,84,86,88,89] •Convenience [12,15,23,31,33,37,38,42,43,48,63,69,75,79,83,84] •Financial [40,48,53,73,84] •Resources [12,26,30,37,40,45,50,62,69,73–75,77,78,80–84]
	***Social opportunity*** Social influence, pressure, norms, conformity, comparisons	•Peers and partners [15,25,33,40,48,51,52,58] •Stigma reduction [46,81] •Friendly language [45] •Relation with provider [35,59,80] •Support [15,34,59,63,86]	•Counselling [30,48,53,62] •Gender of GPs (16, 67) •Judgement (6, 25, 26, 31) •Good relationship with patient (20, 35, 49, 58, 67, 74)	•Community [60,61,63,64] •Destigmatisation [12,55,69] •Gender [12,48,55,82,88] •Support [12,24,45,55,62–64,72]

GP=General practitioner; STI=Sexually transmitted infection

Most of the studies included were conducted in Europe (n = 50), mainly in the UK (n = 28), Australia (n = 7), Canada (n = 7), South America (n = 3) and New Zealand (n = 3). Studies focused on different patient populations, such as young people [12,15, 23–46], men who have sex with men (MSM) [47–55], migrants [53,54,56–65], and female sex workers [66], and on different care providers, such as GPs [67–77], nurses, pharmacists [73,78–81], and others [37,39,55,63,73,82–88]. Only 34 articles provided information on the theoretical model used, with the most common being planned behaviour [15,35,36,40,51,52,58,75,76], the framework approach [28,63,64,67], health belief [55,61,66,89], interpretative phenomenology [41,43,47,59], and grounded theory [72,74,90]. Finally, the main method used to synthesise the information was thematic analysis (Table 1).

Based on the JBI checklist for qualitative research [22], most studies were methodologically robust. All had adequate representativeness of participants and their voices (Question 8); four (5.7%) were unclear as to the congruity between the research methodology and the research question (Question 2); eight (11.4%) were unclear as to the congruity between the research methodology and the methods used to collect data. The question with the lowest score was Question 7 (up to 39 articles did not clearly address the possible influence of the researcher on the research) (Table 2).

The synthesis of the results, organised by the patient, provider, and system levels according to the COM-B model dimensions, is presented in Table 3 (barriers) and Table 4 (facilitators).

## 1. Barriers to the diagnosis of sexually transmitted infections

1A.- **Patient level**

### Capability

The **physical capability** dimension is related to skills, abilities, or proficiencies. Patients showed barriers regarding physical skills, especially for STIs where self-testing is available. For example, MSM had problems with HIV self-testing due to difficulties in using the kit correctly [50], while women had difficulties with self-collection of vulvovaginal swabs for *Chlamydia trachomatis* (CT) testing [15].

**Psychological barriers** are related to knowledge, memory, attention, decision processes, and behaviour regulation.

- *Lack of information/knowledge* about STIs [15,33,35,44–46,52,57,61,69,86,89], their transmission and complications [23,53,62], testing sites and methods [32,36,41–43,55,60,65,91,92], and health system performance [54,58,64,88] were the most common psychological barriers, along with *c*oncerns about *confidentiality* [32,42,44,59,60,62,64,66,73,80,85,89].
- *Negative attitudes* towards testing were seen in certain groups, such as transgender men in relation to HIV (fear of mistreatment) [88], young people (lack of responsibility) [23], people at risk of HIV infection (consequences of a positive result) [50,53,57,60], and injecting drug users (IDU) (negative past experiences) [79]. Negative opinion towards health providers also limited testing [40,51,55]. In addition, *behavioural* barriers were observed [33,64,71,73,77,85,86], namely a feeling of surveillance among students screened for CT and Neisseria gonorrhoeae (NG) (the perception of testing as surveillance or moral judgment can undermine the willingness to seek diagnosis) [33], a reduced need for testing among men [64,71,77], a lack of responsibility among young people [73], and doubts about their right to be tested for HIV among MSM migrants [86].
- *Language barriers.* Finally, language was a common barrier among migrants [12,54,57,58,61,62,65] and people with low education level [76,89], as were *communication* problems among MSM in relation to HIV [28,86].

### Motivation

**Reflective barriers** are related to beliefs about capabilities and consequences, roles, identity, intentions, goals, and optimism.

- *Risk perception* was the most frequent barrier [15,25,27,31,33,38,41,43,46,47,49,53,55,58,59,64,66,92] perfectly expressed by patients (“*It’s better not to know*”) [53]*.* Low risk perception was expressed by young people [15,25,33,38,41,46], migrants [53,58,59,64], and people with sexual risk factors such as MSM and sex workers [29,47,49,55,66].
- *Misconceptions* were highly relevant and related to HIV (conspiracy) [54], monogamy [41,92], traditional medicine [32,65], symptomatic STIs [44], lack of treatment [29], thinking “*I’m sick”* [66], immoral lifestyle [44], medical procedures [32,42,50,52,54,55,60,61,63,64,66,88], and masculinity [32,43]. Finally, *guilt* can lead to denial as a coping mechanism and was observed in screening for CT in young people and for HIV in female sex workers [15,34,46,66].

**Automatic barriers** are related to emotions, reinforcement, and punishment.

- *Embarrassment* [15,24,25,32,33,35,36,38,40,41,44–46,55,56,69,79,86,90,92] and *fear* [12,15,27,29,31,35,39,40,48,49,51–55,60–64,66,70,79,86,88,92] were the most common automatic barriers. People felt ashamed of peers [40] or others [38], of being gay or a MSM [55,56,86], of the associated stigma [33,44], of having to undress in front of the GP [15,25], or of talking about their sexual habits [24,32,36,41,45,46,92].
- *Fear* was related to aversion to invasive procedures [31,43,48,55,86], positive test results [29,40,51,54,55,86,88], judgement by others [35,55,62], deportation [12,53,54,60], disclosure [48,61,62,86], death [27,35,49,53,64,92], social stigma [46,48,53,61,63], testing by indicator conditions (IC) [70], and costs of testing [55].
- *Discomfort* associated with the clinic environment or waiting room [12,42,45,88], the burden of testing [52], or inappropriate locations for testing [85] can also limit screening.

### Opportunity

The main **physical opportunity barriers** were related to *access* [12,28,40,42,43,56–58,61,62,83,88] and *time constraints* [12,36,42–44,60].

- *Access difficulties* were reported by female students [40], young people [42], MSM [12,28,56,88], low-qualified workers [43], and migrants [58,62].
- *Time constraints* were identified as lack of time for consultations [36], limited opening hours of clinics [12,42], difficulties in getting time off [43,44] or medical appointments [12,40,43–45,47,48,57,66,79,86], and concerns about the time commitment associated with increasing the frequency of HIV testing [60].
- *Financial* [43,46,47,53,57,64–66,86] *and geographical* [12,46,54,57,66] issues were also physical barriers for unqualified workers [43], rural women [46], MSM [12,47,86], female sex workers [66], and migrants [53,57,64,65].

The social aspect of the opportunity domain has to do with social influence, pressure, norms, conformity, and comparisons. **Social opportunity barriers** were the most common for patients.

- *Discrimination/stigma* around STIs and HIV was particularly relevant [12,15,25,28,31,38,39,41,42,49,51,54–57,60–62,64,65,70,83,85,86,90] and reported by different groups such as migrants [54,57,60–62,64,65], MSM [12,28,49,51,85], sex workers [55,56,86], and young people [15,26,41,42].
- *Culture and religion* were also important [36,53,54,61,62,64,65,69,72,92], especially for migrants [53,54,61,62,64,72] and for mass HIV screening [92].
- *Disclosure* aspects [15,26,40,42,51,55,79,83,88] can be problematic for MSM [51,55,88] and for young people with risky sexual behaviour [15,25,40,42,83].
- *Lack of social support* was a barrier for voluntary HIV testing [43], migrants [57,62,64], MSM [47,86], and transgender people [48]. Other social barriers were related to *gender* [12,42,48,65,71,88], particularly for transgender people (few trans-friendly resources or transphobic attitudes) [12,42,48,88] and African, Caribbean, and Black (ACB) communities who considered the gender of the GP as a barrier [65]. Other less common social barriers included c*oncerns* about different aspects [24,34,45,62,85], *feeling judged* by professionals [25,27,40,46,70,79], *negative comments from peers* [23,33], s*ocial norms* [28,55], and *poor provider-patient relationship* [51,57,62,74,93].

In summary, the main barriers at patient level were lack of information/knowledge, confidentiality, negative attitudes and language (Capability); risk perception, misconceptions, embarrassment and fear (Motivation); and access, time constraints, discrimination/stigma and disclosure aspects (Opportunity). These barriers were common for both, HIV and other STIs.

1B.- **Provider level**

### Capability

The main **physical capability barriers** for providers were:

- *Lack of expertise* in STIs [34,40,61,63,68,75], as reported both by themselves [68,75] and by patients (lack of professionalism of GPs) [34,40,61,63].
- *Self-testing* for HIV and CT can be a barrier for some providers because, although it takes pressure off the system, it can lead patients away from a holistic approach, with potential health consequences [50,69].
- *Skill barriers* were reported by pharmacists showing difficulties in collecting adequate samples for HIV PoC testing [83].

**Psychological barriers** were the most common for providers that acknowledged:

- *Lack of knowledge* about testing and the benefits of screening [30,68,69,74,75], HIV or acquired immunodeficiency syndrome (AIDS)-defining illnesses [27], time to test [12,70,74], applicability of HIV testing by ICs [70], training to provide results [83,84], awareness of pharmacy testing [23], relevance of pre-test counselling [72], and services for MSM [65,72].
- *Communication problems* [68,69,71,74,75,77], *language* barriers [68,72,76], and a*ttitude aspects*. GPs reported personal discomfort with HIV-related issues [51,68] and negative attitudes towards screening by nurses [80], and receptionists expressed hostility [64,79]. Physicians were also concerned about *patients’ feelings* when offered screening (*e.g.,* if they felt offended or stigmatised) [68–70,72,75–77]*.* Finally, the confidentiality or privacy needs of people diagnosed with HIV/STIs were also expressed by providers [42,73,81].

### Motivation

The only **reflective barrier** for providers was the low*-risk perception* due to the asymptomatic nature of STIs, especially among young people and men in relation to HIV [77,88].

**Automatic barriers** were *missed opportunities to engage in the system, e.g.,* due to HIV self-testing in MSM [50], the need for follow-up [73,86], or contact tracing [74] due to confidentiality concerns and the frustration feeling of providers that they do not have enough resources or cannot recruit new staff to meet the needs of STI services [73].

### Opportunity

The **physical opportunity barriers** were mainly related to *time constraints.* Lack of time for consultations, pressure, and workload constraints were frequently reported by providers [62,67,68,70,72–77,80,81] and patients [15,34,35,44].

As with patients, **social opportunity barriers** were also the most common among providers:

- *Discrimination/stigma* [12,42,56,57,60,64,81] was very relevant. MSM reported stigmatising barriers from providers related to gender [12], homophobia and heterosexism [42], and HIV health promotion [56]. Migrants and ACB communities reported racism and stigmatising attitudes from GPs [57,60,64]. Healthcare staff also expressed stigmatising views about HIV testing [81].
- *Cultural issues* were also common [40,60,62,64,72,77]. ACB communities expressed racism and lack of cultural competence within health systems [60]. Refugee women acknowledged lack of HIV-specific services for multicultural populations [62]. Migrants and sub-Saharan African migrants reported insinuations about fidelity when being offered a test and a lack of culture-sensitive counselling [64,72], while GPs expressed difficulties for men in consulting a female GP [77]. Finally, professional judgement was perceived by the ACB population in HIV testing and by rural women in CT testing [46,60,70].

In short, the main barriers at provider level were the lack of expertise or knowledge and communication problems (Capability); low risk perception (Motivation); and time constraints, discrimination/stigma and cultural issues (Opportunity). These barriers were also shared by HIV and other STIs.

1C.- **System level**

### Capability

**Physical capability barriers**. *Integrated care pathways* can be a barrier for providers due to their importance in improving the standard of care for HIV patients [63*].*

**Psychological capability barriers.** The most common were related to:

- *Information issues* [32,40,46,53,56,62,65,83,86,92]*,* such as inaccessible information about screening services [32,65,92] or continuum of care following a reactive test by pharmacists [83], perceptions of reduced effectiveness of voluntary counselling and testing (VCT) by practitioners [62], and lack of transparency of procedures [53].
- *Confidentiality* was particularly important for young people [34,35,64,75].
- The perception of *HIV/AIDS as an exceptional disease* made it difficult for GPs to integrate HIV testing into routine care [72]. GPs also reported that indicator condition testing (IC-guided) is too long and not applicable in PC [70].
- The lack of *language resources* for migrants can also be problematic for providers [57,62,72].

### Motivation

Providers reported a *lack of awareness* of alternative testing sites [43], STIs among health professionals [64], services for sex workers, and the confidential and free-of-charge nature of sexual health services [55].

### Opportunity

**Physical opportunity barriers** were particularly prevalent at the system level, specially referred to:

- *Financial issues* related to transport costs, public health measures, co-payment or lack of funding for organisations working with migrants [25,31,32,53,57,62,64,66,69,70,74,82,86,92]*.*
- *Lack of resources*. GPs expressed incompatibility of HIV testing with PC [68], limited capacity [27,42,67,73,88], insufficient training or staffing [39,74,75], and system strain [15].
- *Time constraints* were reported as system pressure, professional workload, rigid scheduling of medical appointments, and difficulty in getting an appointment [12,15,44,61,68,69,72–74].
- *Access problems* were also important at the system level, with difficulties in getting appointments [15,30,31], long waiting times [12,27,40,61], and a referral process for HIV PoC testing in community pharmacies particularly problematic for migrants [83].

**Social opportunity barriers** were reported as:

- *Lack of support* for partner notification [69] and from religious leaders, social networks [57], and psychosocial services [64]. Providers did not feel supported by the whole team [75], and African communities expressed a lack of trust in the health system [61].
- *Discrimination/stigma* was relevant for MSM and African migrants who expressed the need for STI testing to take place in an inclusive, culturally safe, and non-stigmatising clinical setting [12,57,64,81].
- *Gender* issues were reported by young people who described clinics as ‘feminised’ spaces [42], and by transgender men who highlighted the need for clinics to be inclusive for all people, regardless of their sex assigned at birth [12], and expressed a gap between trans-inclusive policy and practice [88].
- S*creening by non-healthcare professionals* [26,75,82], the risk of jeopardising the client relationship with the pharmacist [78], and the lack *of privacy in the reception area* [34,67] were additional social opportunity barriers*.*

In brief, the main barriers at system level were information issues, confidentiality (Capability); lack of awareness (Motivation); and financial issues, lack of resources and time constraints (Opportunity). These system barriers were also shared by HIV and other STIs.

## 2. Facilitators in the diagnosis of sexually transmitted infections

2A.- **Patient level**

### Capability

Capability barriers were expressed as:

- *Never having been tested* motivated the screenings for HIV PoC test users [83].
- *Awareness.* It is essential to raise awareness of the need for education at young ages, the asymptomatic nature of some STIs, confidentiality, and the benefits of screening [25,28,34,45,54].
- *Confidentiality.* Ensuring confidentiality [12,37,40,59,62,83,89] was critical for MSM [12], young women [40], migrants [59,62], and internet-based screening [37,89]. Pharmacists were facilitators for PoC test users due to confidentiality [83].
- *Information.* The facilitating role of information was related to explaining the procedure as well as the benefits of testing and follow-up steps and to open communication about sexual health issues [6,9,14,18,34,45].
- *Normalisation of testing* [31,78] was particularly important for young people [41] and for HIV screening [88].

### Motivation

The most common **reflective motivation facilitators** were:

- *Risk* awareness [12,25,33,43,46,55,59,66,86]*.* Those who perceive themselves as being at low risk may use testing as a way to confirm their negativity [46]. Conversely, individuals who perceive themselves as being at high risk of STIs may be more likely to get tested, including patients taking pre-exposure prophylaxis [12], sex workers [55], people who know someone with a STI, and women who want to protect their fertility [25]*.*
- The *responsibility for partner protection and contact tracing* [25,28,55,66,74,86,88] and the need to *reduce the stress and anxiety,* expressed as the need for “peace of mind”, “being happy with screening”, “positive impact of taking the test”, “prompt reassurance to relieve anxiety”, or “wanting to stop worrying” [43,45,47,51,66,90], were also very common.
- The *availability of treatment* was a facilitator for African migrants [61] and female sex workers [66].
- *Trust* in partners, medical staff, and STI clinics, or between lay providers and end users was also reported by patients [29,36,44,55,61,84].

The **automatic motivation facilitators** were related to:

- *Perceived vulnerability*, such as having unprotected sex, feeling vulnerable to infections, having an increased number of sexual partners, changing a sexual partner, and being in a “transitional period” [12,25,28,52,83,88].
- *Work-related factors* (STI testing required by an employer) [25,66]
- *Emotional preparation* for visits, reported by IDUs undergoing testing [79].

### Opportunity

In the **physical opportunity dimension** the facilitators reported were:

- *Facilitating access* by reducing waiting lists or increasing the availability of trusted providers or checkpoints was very important for patients [40,42,70,79,83,87,88].
- *Time constraints,* such as avoiding unnecessary visits, reducing waiting times, speeding up referrals of positive people to HIV services, and getting rapid results [12,40,43,47,63,83,84,86,89].
- *Convenience* aspects were reported for CT pharmacy testing (long opening hours) [23], CT non-medical screening [38,45], postal sample kits [40], HIV rapid testing [43,59], free-of-charge self-sampling test service for CT/NG [89], discreet environment for rapid HIV test checkpoint [84], and self-testing [11,23,30,46,49,59].
- *Financial factors,* including the use of incentives and free-of-charge home self-testing for CT and NG [67,89], free-of-charge newer approaches such as provider-initiated HIV testing and counselling and self-testing [94], and the availability of free treatment in clinics [40].

**Social opportunity facilitators** were mainly related to:

- P*eers and partners* who can facilitate testing [15,25,33,40,48,51,52,58] and friends and parents who provide *support* [15,34,59,63,86].
- *The relationship with the provider* is also important, as some patients felt more comfortable with a doctor they knew, and women with female providers [35,59,80]. Increased knowledge and routine testing reduced the stigma [46,81]*.*
- *Language*. Patients enjoyed the ‘light-hearted’ approach and the use of ‘friendly’ language [45].

In summary, the main facilitators at patient level were awareness of benefit of screening, ensuring confidentiality to patients, and the open information about sexual health issues (Capability); risk awareness, responsibility and trust with partners and perceived vulnerability (Motivation); and facilitating access, rapid tests and results, self-testing and support from peers and partners (Opportunity). As with barriers, patient-level facilitators are common to HIV and other STIs.

2B.- **Provider level**

### Capability

Providers expressed the need for expertise and **physical** *technical skills* to manage sexual health and testing [74,83], and young professionals can be very useful in the introduction and distribution of CT testing kits [34]. Closely related, **psychological facilitators** were the need for counselling and *training* to discuss on sexual health [15,23,31,32,35,36,40,59,69,72,80,83,84]*, information* to answer patients’ questions [12,15,39,40,55,60,70,75,81], and the *normalisation of STI testing,* as a public health intervention [12,26,70,81].

### Motivation

In the **reflective motivation** dimension, providers mentioned *responsibility* or moral obligation to others [15] and *trust* in the patient-provider relationship [61,72,84].

**Automatic motivation facilitators** were *incentive payments* [69], *positive reinforcement* [15], and *patient empowerment* by involving practice nurses in testing [80].

### Opportunity

**Physical opportunity** facilitators were *convenience* of home testing [44], use of *appropriate language* at the PoC [62], *counselling* for sexual issues, cervical screening, or contraception [76], and *referral* of positive individuals to other health services [63,79].

The most common **social opportunity** facilitators were maintaining a good *patient-physician relationship* [23,38,52,61,70,77] based on *non-judgmental* interactions [6,28,29,34] and *counselling, e.g.,* about *a*nonymous HIV testing clinics, pretest discussions, and a p*roactive approach* to HIV [30,48,53,62]. Finally, the *gender of GPs* is important when asking about sexual health issues [17,70].

At provider level more frequent facilitators were information and training on sexual health (Capability); and a good patient-physician relationship with non-judgmental interactions (Opportunity) and both facilitators were observed for HIV and other STIs,

2C.- **System level**

### Capability

Among the **physical capability facilitators**, the need for *training* was reported for both patients and professionals regarding official recommendations, STI awareness, guidelines for VCT counsellors, workshops, and online medical education [15,62,63,67–69,72,74,75,77,80,81,83,84].

**Psychological capability facilitators** were mainly related to *education and information issues.* The importance of proactive sex education at young ages, STI prevention, and the usefulness of social media awareness campaigns to change the perception of free testing as a substitute for condoms, as well as peer education for HIV-positive men, were highlighted [15,25,32,33,40,44,49,60–63,69,84,89]*. Information* about personal responsibility, benefits of testing, testing sites, home testing, self-sampling kits, online information, and posters and leaflets in multiple languages were emphasised [12,15,44,55,58,60,61,64,74,75,85,89].

Other facilitators included the use of *alternative methods* (non-invasive, self-testing, alternative staff, internet-based screening, mobile VCT or “health bus” for vulnerable groups such as MSM) [15,34,45,55,71,74,82,85] and *ensuring confidentiality* [24,37,45,64,71,83].

### Motivation

The use of *incentives*, although not necessarily money, was reported as a motivation facilitator for CT and NG screening [33].

### Opportunity

The most important **physical opportunity facilitators** were related to:

- *Access to the system.* The following themes were mentioned: improving appointments, reaching vulnerable populations, availability of screening kits, referrals to sexual health services, and testing in non-profit organisations or non-medical settings [12,23,24,30,33,37,41,45,49,50,62–64,75,79,82,84,86,88,89]*.*
- *Adequate resources.* Closely related with the access to the system. STIs should be prioritised with national screening programmes; GP overload should be reduced with shorter waiting times, longer consultations, and greater involvement of nurses in testing. Expert care should be multidisciplinary with the involvement of specialist staff in case of positive results [12,26,30,37,40,45,50,62,69,73–75,77,78,80–84].
- *Convenience issues* were common and related to the location of testing sites, invitation letters, flexible appointments, reminders, evening and weekend testing, inclusive clinics for all genders, and private areas in the pharmacies [12,15,23,31,33,37,38,42,43,48,63,69,75,79,83,84].
- *Financial* facilitators were linked to free testing, free treatment available in clinics, and elimination of fee-for-service billing [40,48,53,73,84].

*Supporting* patients was the main **opportunity psychological facilitator.** The need to develop specific policies, provide language services, use patient-centred language, and have peer volunteers in clinics and telephone helplines was reported [12,24,45,55,62–64,72]. The *community* can facilitate testing by increasing education and awareness, community outreach activities, the role of leaders, mobilisation with institutions, and engagement with faith-based organisations [60,61,63,64]. *Destigmatisation and gender issues* were also common. In this regard, issues such as the screening of women by female professionals, specialised clinics for transgender people, promotion of sexual health services for MSM, integration of testing with gender-affirming treatment, and posters using transgender language were mentioned [12,48,55,69,82,88].

At system level common facilitators were medical and sexual education and information, and the use of alternative methods (Capability); and factors facilitating the access to the system, with provision of adequate resources, and support for patients with destigmatization actions (Opportunity), No specific differences were observed between HIV and other STIs regarding these types of facilitators.

## Discussion

This SR addresses barriers and facilitators to HIV/STI diagnosis in PC, following a theoretical behavioural model that allows the design of effective intervention strategies.

### Main findings

According to our findings, the main barriers for patients and professionals are consistent across HIV and other STIs. The results often revealed barriers for patients and professionals around stigma and feelings of judgement or shame, as well as a lack of knowledge or practice in sexual interviewing. Another common barrier was confidentiality, which was also highlighted as a structural barrier, as were access to care, especially for migrant populations, lack of information about HIV/STIs, and the need to use languages that patients can understand. An additional shared and recurring theme was the need to use minimally invasive tests and to standardise screening in the at-risk population, particularly in small areas or where there are no STI-specific clinics to provide these services. In addition, the SR identified several key facilitators to enhance the diagnosis of HIV/STIs, such as the use of minimally invasive sampling methods, ensuring confidentiality, standardization of tests, guarantee fast and efficient entry points, and facilitate professional training. All these factors were recurrent in the included studies and are consistent with other SRs conducted on specific STIs, such as chlamydia in young people [17,95].

Distinguishing HIV from other STIs enables the identification of the specific and complex barriers associated with HIV diagnosis. This approach supports the design of targeted interventions that address psychological stigma, linkage to lifelong care, service integration gaps, and policy-related differences, without conflating these with the broader or distinct barriers that affect other STIs. Such differentiation increases the accuracy and contextual relevance of findings, thereby informing more effective public health strategies aimed at the unique diagnostic challenges of each condition. However, the results of this SR did not show important differences in the types of barriers and facilitators between HIV and other STIs, as most of the identified factors appeared to be applicable to both.

On the other hand, the use of the COM-B model allowed us to categorise these barriers and facilitators into different dimensions of behaviour for each of the actors involved in STI diagnosis. It is important to underline that the same barrier or facilitator can impact on different dimensions of the COM-B model because human behaviour is influenced by a complex interplay of psychological, physical and social factors. Barriers and facilitators also have a multifaceted nature with multiple dimensions and reciprocal influences. Lastly, the environment and social context can amplify or mitigate the impact of barriers and facilitators on different dimensions. The theoretical understanding of behaviour using this model, as well as the interaction between factors and domains is essential to determine the changes to be implemented in order to achieve the behavioural goal and the intervention functions that are likely to be effective in bringing about these changes [16].

### Implications for practice

Although the literature and clinical guidelines recommend opportunistic screening in asymptomatic, sexually active patients under 30 years of age [2], there are few studies that address general population screening in PC. Regarding sexual interviewing, the available data are limited, showing that most patients would feel comfortable if their PC physician asked them about their sexual practices in the consultation room [96,97], and often refer to specific groups [98]. In addition, integrating HIV/STI screening into routine clinical practice and training professionals in sexual interviewing skills are key to improving diagnosis [5] and can be easily implemented in our PC settings. Addressing the perception of risk associated with certain sexual behaviours and ensuring the availability of accessible and rapid pathways with minimally invasive sampling methods are critical. In fact, scales have been developed to estimate risk, although they are not clearly implemented in daily clinical practice [99,100]. Professionals also emphasised the need for support to adopt a proactive attitude in daily clinical practice. Finally, some macro-level (system) facilitators for diagnosis include integrating these pathways into the community, conducting destigmatisation campaigns, and supporting the most vulnerable groups. Similarly, the implementation of the main facilitators obtained (minimally invasive sampling methods, confidentiality, standardization of testing, fast and efficient entry points and professional training), facilitates the accessibility and efficiency of HIV/STI screening, ultimately contributing to improved public health outcomes.

PC physicians play an essential role in STI screening, with some studies showing that up to half of cases are reported by PC clinics, but there is still room for improvement. STI screening should be an integral part of a comprehensive PC visit. Although PC providers are the frontline physicians for many adolescents and young adults and are in an ideal position to screen this population for STIs [5,101,102], many do not take full advantage of this opportunity. Every visit to a GP is an opportunity to provide sexual health care. Because adolescents and young adults account for so much of the HIV/STI burden, more effective strategies are needed to identify these often-hidden infections in young patients. Normalising routine STI screening can lead to earlier detection and treatment, reduced transmission, and better protection of patients’ reproductive health [5].

The findings from this SR can inform policy and practice in several ways: addressing stigma and judgement, enhancing knowledge and skills, ensuring confidentiality, improving access to care, using minimally invasive tests, standardizing screening, facilitating fast and efficient entry points, and professional training. By addressing these areas, policymakers and healthcare professionals can work together to overcome the barriers and enhance the facilitators, ultimately improving the diagnosis and management of HIV/STIs in PC settings. The implementation of these actions in PC is aligned with the strategies published by the WHO to end STIs as public health concerns by 2030 [103].

### Strengths and limitations

This study has strengths and limitations. The strengths include the number of articles included, the approach to HIV and all STIs that can be diagnosed in PC, the inclusion of the general population and specific groups, and the use of a behavioural model to categorise barriers and facilitators in order to design intervention strategies from the perspective of the different actors involved in the management of STIs in PC (patients, professionals, and system). The inclusion of HIV and all STIs in the review is a strength compared to other reviews that have only included specific conditions such as CT infection [17,95], taking into account the need for a broader view of the problems faced by patients with any type of STI when seeking care.

Among the limitations, we only included studies conducted in countries with a health system that can be extrapolated to Spain, where the GP acts as a gatekeeper for access to specialist services and general practice is publicly funded. This approach may limit the external validity of our findings in different contexts. However, cultural and health system factors, particularly in relation to access to care and primary and secondary prevention, play a crucial role in the development of effective intervention strategies for STI diagnosis [104,105]. Consequently, local factors are important when designing intervention strategies for the diagnosis of HIV/STIs in PC because they directly influence the effectiveness, feasibility, and equity of these interventions. Moreover, the WHO strategy for ending STIs as a public health problem for the period 2022–2030 specifically underlines the importance of defining and implementing research agendas at national and regional level to adapt actions to the local epidemiological and health system contexts [103]. Therefore, intervention strategies must be context-specific to maximize their public health impact and ensure equitable access to diagnosis and care [8].

Therefore, although the exclusion of studies from non-comparable healthcare systems limits the external validity of these findings, this approach aligns with WHO recommendations to adapt STI research to local contexts, prioritizing actionable insights for the target setting over broad generalizability. The deliberate focus on public systems like Spain’s enhances internal validity and practical relevance for intervention design, trading off wider applicability for context-specific efficacy. Future research could address this limitation by incorporating multi-country meta-analyses or transferability frameworks, as suggested in global STI agendas [106].

### Conclusions

In conclusion, this SR addresses the diagnosis of any type of STI in PC from the COM-B model perspective. By applying this model, the SR advances a theoretically grounded understanding of how behavioral, structural and contextual factors intersect to shape STI diagnostic practices. The findings underscore the importance of developing multifaceted, contextually adapted interventions that integrate routine screening into PC, normalize sexual health discussions, and enhance professional competence.

Addressing these barriers while strengthening identified facilitators is essential to improving early detection, reducing transmission, and safeguarding reproductive health. Furthermore, aligning PC strategies with the WHO targets for STI elimination by 2030 offers a clear framework for policymakers and practitioners. This review therefore contributes to the evidence base by highlighting actionable pathways to optimize STI diagnosis in PC and by reinforcing the central role of general practice in advancing sexual health at the population level.

## Supporting information

S1 File Checklist. PRISMA 2020 checklist. (DOCX)

S2 File Search strategy and excluded articles. (DOCX)

S3 File All retrieved articles. (XLSX)

S4 File Results of individual studies. (DOCX)

## Abbreviations

ACB: African, Caribbean, and Black
AIDS: Acquired immune deficiency syndrome
COM-B model: Capability, Opportunity, Motivation model of Behavior
CT: *Chlamydia trachomatis*
GP: General practice/practitioner
HIV: Human immunodeficiency virus
IC: Indicator condition
IDU: Injecting drug user
JBI: Joanna Briggs Institute
MSM: Men who have sex with men
NG: *Neisseria gonorrhoeae*
PC: Primary care
PoC: Point-of-care
SR: Systematic review
STI: Sexually transmitted infection
UK: United Kingdom
USA: United States of America
VCT: Voluntary counselling and testing
