# Supplementary material for: Barriers and facilitators to the diagnosis of HIV and other STIs in primary care within publicly funded healthcare systems: A systematic review of qualitative studies
Source: PLoS One. 2026 Feb 5;21(2):e0341919. doi: 10.1371/journal.pone.0341919 (PMC12875586; doi:10.1371/journal.pone.0341919)
Supplement: S2 File — (DOCX) [file pone.0341919.s002.docx]

**Supplementary material**

**Table S2_1**. Search strategy in Pubmed.

| # | Search terms | References |
| --- | --- | --- |
| #1 | ("Sexually Transmitted Diseases"[MeSH Terms] OR "sexually  transmitted*"[Text Word] OR "venereal disease*"[Text Word] OR  "chlamydia*"[Text Word] OR "syphilis*"[Text Word] OR  "gonorrhea*"[Text Word] OR "HIV"[Text Word] OR "Mycoplasma  genitalium"[MeSH Terms] OR "mycoplasma genitalium*"[Text  Word]) | 571629 |
| #2 | ("Primary Health Care"[MeSH Terms] OR "primary health  care*"[Text Word] OR "primary healthcare*"[Text Word] OR  "primary care*"[Text Word] OR "Public Health"[MeSH Terms] OR  "public health*"[Text Word] OR "community"[Text Word]) | 9902782 |
| #3 | ("Diagnosis"[MeSH Terms] OR "diagnos*"[Text Word] OR  "screening*"[Text Word] OR "test*"[Text Word]) AND ("barriers and  facilitators"[Text Word] OR "barrier*"[Text Word] OR  "facilitator*"[Text Word]) AND ("Qualitative Research"[MeSH  Terms] OR "qualitative*"[Text Word]) | 11675 |
| #4 | #1 AND #2 AND #3 | **1207** |

**Table S2_2**. Search strategy in Embase.

| # | Search terms | References |
| --- | --- | --- |
| #1 | ((sexually AND transmitted AND ('disease'/exp OR disease) OR sexually) AND transmitted AND ('disease'/exp OR disease) OR sexually) AND transmitted* OR 'venereal disease*' OR 'chlamydiae'/exp OR 'chlamydiae' OR chlamydia* OR 'syphilis'/exp OR 'syphilis' OR syphilis* OR 'gonorrhea'/exp OR 'gonorrhea' OR gonorrhea* OR 'human immunodeficiency virus'/exp OR 'human immunodeficiency virus' OR hiv* OR 'mycoplasma genitalium'/exp OR 'mycoplasma genitalium' OR 'mycoplasma genitalium*' | 751203 |
| #2 | ('primary health care'/exp OR 'primary health care' OR 'primary health care*' OR 'primary healthcare*' OR 'primary care*' OR 'public health'/exp OR 'public health' OR 'public'/exp OR public) AND health* OR 'community'/exp OR 'community' OR community* | 3280094 |
| #3 | #1 AND #2 | 162727 |
| #4 | 'diagnosis'/exp OR 'diagnosis' OR diagnos* OR 'screening'/exp OR  'screening' OR screening* OR test* | 16012374 |
| #5 | #3 AND #4 | 82281 |
| #6 | 'barriers and facilitators' OR barrier* OR facilitator* | 588561 |
| #7 | 'qualitative research'/exp OR 'qualitative research' OR qualitative* | 524400 |
| #8 | #5 AND #6 AND #7 | 1314 |
| #9 | #8 AND([article]/lim OR [article in press]/lim OR [data papers]/lim  OR [editorial]/lim OR [letter]/lim OR [note]/lim OR [review]/lim OR  [short survey]/lim) | 1095 |
| #10 | #9 AND [embase]/lim NOT ([embase]/lim AND[medline]/lim) | 101 |
| #11 | #9 AND [embase]/lim NOT ([embase]/lim AND [medline]/lim) AND  ([cochrane review]/lim OR [systematic review]/lim OR [meta  analysis]/lim) | 6 |
| #12 | #9 AND [embase]/lim NOT ([embase]/lim AND [medline]/lim) AND  ([randomized controlled trial]/lim OR 'controlled clinical trial'/de) | 4 |
| #13 | #9 AND [embase]/lim NOT ([embase]/lim AND [medline]/lim) AND  ('cohort analysis'/de OR 'longitudinal study'/de OR 'observational  study'/de OR 'prospective study'/de OR 'retrospective study'/de) | 10 |
| #14 | #11 OR #12 OR #13 | 19 |
| #15 | #10 NOT #14 | **82** |

**Table S2_3.** Search strategy in the Cochrane Library.

| # | Search terms | References |
| --- | --- | --- |
| #1 | MeSH descriptor: [Sexually Transmitted Diseases]  explode all trees | 19071 |
| #2 | (sexually transmitted*):ti,ab,kw | 3019 |
| #3 | (venereal disease*):ti,ab,kw | 68 |
| #4 | (Chlamydia* OR syphilis* OR gonorrhea* OR HIV*):ti,ab,kw | 33739 |
| #5 | MeSH descriptor: [Mycoplasma genitalium] explode all trees | 31 |
| #6 | (mycoplasma genitalium*):ti,ab,kw | 67 |
| #7 | #1 OR #2 OR #3 OR #4 OR #5 OR #6 | 36794 |
| #8 | MeSH descriptor: [Primary Health Care] explode all trees | 10404 |
| #9 | (primary health care* OR primary healthcare* OR primary care* OR community*):ti,ab,kw | 168315 |
| #10 | MeSH descriptor: [Public Health] explode all tres | 603989 |
| #11 | (public health*):ti,ab,kw | 25316 |
| #12 | #8 OR #9 OR #10 OR #11 | 726027 |
| #13 | #7 AND #12 | 18157 |
| #14 | MeSH descriptor: [Diagnosis] explode all tres | 446376 |
| #15 | (diagnos* OR screening* OR test*):ti,ab,kw | 701118 |
| #16 | #14 OR #15 | 932040 |
| #17 | #13 AND #16 | 12259 |
| #18 | ("barriers and facilitators" OR barrier* OR facilitator*):ti,ab,kw | 27700 |
| #19 | #17 AND #18 | 790 |
| #20 | ("qualitative research"):ti,ab,kw | 4228 |
| #21 | (qualitative*):ti,ab,kw | 22870 |
| #22 | #20 OR #21 | 22870 |
| #23 | #19 AND #22 in Trials | **184** |

**Table S2_4**. Excluded studies and reasons for exclusion.

| # | Author, year | Reason for exclussion |
| --- | --- | --- |
| 1 | Adedimeji_2010 (1) | Retracted from publication |
| 2 | Alvim_2023 (2) | Article in Portuguese |
| 3 | Baidoobonso_2013 (3) | Risk factors for HIV infection |
| 4 | Bates_2007 (4) | Study conducted in US |
| 5 | Beougher_2015 (5) | Study conducted in US |
| 6 | Blondell_2015 (6) | Systematic review |
| 7 | Brawner_2018 (7) | Study conducted in US |
| 8 | Brookfield_2020 (8) | Systematic review |
| 9 | Brown_2023 (9) | Screening assessment study |
| 10 | Burchell_2019 (10) | Protocol |
| 11 | Calin_2007 (11) | Disclosure and social support of HIV+ persons |
| 12 | Cassidy_2018 (12) | Predictors of sexual health service use |
| 13 | Chorba_2004 (13) | Systematic review |
| 14 | Collyer_2018 (14) | Promoting sexual health, not ITS screening |
| 15 | Conserve_2017 (15) | Systematic review |
| 16 | Corker_2022 (16) | Acceptability of a diagnostic test method, not the barriers and facilitators to screening |
| 17 | de Munnik_2017 (17) | Sexual risk behaviour not ITS screening |
| 18 | Ekstrand_2011 (18) | Barriers for safe sex not for screening |
| 19 | Flowers_2000 (19) | No criteria for inclusion |
| 20 | Flowers_2001 (20) | No criteria for inclusion |
| 21 | Freeman_2009 (21) | Posters and brochures for the promotion of chlamydia screening |
| 22 | Gesink_2020 (22) | Sexual health knowledge/attitudes of MSM, not barriers/facilitators to screening |
| 23 | Gkatzidou_2015 (23) | Design of mobile-based sexual health interventions |
| 24 | Gudka_2013 (24) | No criteria for inclusion |
| 25 | Ho_2017 (25) | Protocol |
| 26 | Horwitz_2022 (26) | How to improve stigma in subjects with various pathologies, including STIs (without differentation) |
| 27 | Kesten_2017 (27) | Abstract |
| 28 | Kesten_2019 (28) | HIV education and its impacts on HIV testing |
| 29 | Kielly_2018 (29) | Protocol |
| 30 | King_2013 (30) | Health care services in Russia |
| 31 | Kiridaran_2022 (31) | Sexual health services, not barriers and facilitators of STI screening |
| 32 | Koester_2016 (32) | Study conducted in US |
| 33 | Larcombe_2023 (33) | Aboriginal people |
| 34 | Leblanc_2016 (34) | Meta-Synthesis |
| 35 | Leddy_2019 (35) | Scoping review |
| 36 | Leidel_2015 (36) | Systematic review |
| 37 | Leitinger_2018 (37) | HIV prevention programme |
| 38 | Lorch_2015 (38) | Opinion of GP about role of PN in chlamydia screening |
| 39 | Lorenc_2011 (39) | Systematic review |
| 40 | Lui_2018 (40) | Systematic review |
| 41 | Manirankunda_2021 (41) | Impact of HIV diagnosis in the lives of sub-saharan migrant living in Belgium |
| 42 | McDonagh_2017 (42) | Protocol |
| 43 | McDonagh_2018 (43) | Systematic review |
| 44 | Metcalfe_2015 (44) | Psychological support and strategies for disclose of sexual orientation of MSM |
| 45 | Middleton_2020 (45) | Design and testing of a SMS intervention for HIV prevention |
| 46 | Mocelin_2023 (46) | Article in Portuguese |
| 47 | Mullens_2019 (47) | Satisfaction with a new POCT |
| 48 | Nanhoe_2018 (48) | Partner notification |
| 49 | Natoli_2015 (49) | Aboriginal people |
| 50 | Padovese_2021 (50) | Prevalence and risk behaviour of STIs |
| 51 | Paiva_2011 (51) | Disclosure of HIV+ serostatus to partners |
| 52 | Palinkas_2014 (52) | Couples-based program of sexual and drug risk behaviours |
| 53 | Patterson_2012 (53) | Protocol |
| 54 | Ransom_2005 (54) | Study conducted in US |
| 55 | Reed_2015 (55) | Partner notification of adolescent patient |
| 56 | Reed_2017 (56) | Study conducted in US |
| 57 | Ricketts_2016 (57) | Intervention study |
| 58 | Ruffinen_2015 (58) | Aboriginal people |
| 59 | Schaffer_2013 (59) | Article in German |
| 60 | Seguin_2018 (60) | Cost-effectiveness of a intervention to enhance HIV testing |
| 61 | Snelgrove_2012 (61) | Physician perceptions of barriers to healthcare provisions for transgender patients. |
| 62 | Spicer_2011 (62) | Access to the health system and not diagnosis. |
| 63 | Theunisen_2014 (63) | Diagnosis in the couple. |
| 64 | Vermandere_2021 (64) | Partner notification |
| 65 | Williams_2017 (65) | Study conducted in US |
| 66 | Witzel_2016 (66) | Evaluation of a specific technique. |
| 67 | Witzel_2017 (67) | Evaluation of a specific technique. |
| 68 | Wood_2018 (68) | Parnert treatment |
| 69 | Worthington_2003 (69) | No inclusion criteria |
| 70 | Wright_2021 (70) | Access of trans people to the health system |
| 71 | Xu_2023 (71) | Scoping review |
| 72 | Yeung_2015 (72) | Narrative review |
| 73 | Young_2022 (73) | Evaluation of a specific technique. |
| 74 | Zapata_2023 (74) | No criteria for inclusion |

**REFERENCES**

1. Adedimeji A. To tell or not to tell: Managing HIV/AIDS disclosure in a low-prevalence context. Sahara j. 2010;7(1):16-23.

2. Alvim FLK, de Jezus SV, da Silva AI, Leão AC, Zambonin F, Maciel ELN, et al. Addressing HIV/AIDS and syphilis in Venezuelan migrant women from the perspective of health managers in the North of Brazil. Revista Panamericana de Salud Publica/Pan American Journal of Public Health. 2023;47(1).

3. Baidoobonso S, Bauer GR, Speechley KN, Lawson E. HIV risk perception and distribution of HIV risk among African, Caribbean and other Black people in a Canadian city: mixed methods results from the BLACCH study. BMC Public Health. 2013;13:184.

4. Bates CJ, Singer M, Needle R, Trotter RT. The RARE model of rapid HIV risk assessment. J Health Care Poor Underserved. 2007;18(3):16-33.

5. Beougher SC, Bircher AE, Chakravarty D, Darbes LA, Mandic CG, Neilands TB, et al. Motivations to test for HIV among partners in concordant HIV-negative and HIV-discordant gay male couples. Arch Sex Behav. 2015;44(2):499-508.

6. Blondell SJ, Kitter B, Griffin MP, Durham J. Barriers and Facilitators to HIV Testing in Migrants in High-Income Countries: A Systematic Review. AIDS Behav. 2015;19(11):2012-24.

7. Brawner BM, Jemmott LS, Wingood G, Reason J, Mack N. HIV/STI Prevention Among Heterosexually Active Black Adolescents With Mental Illnesses: Focus Group Findings for Intervention Development. J Assoc Nurses AIDS Care. 2018;29(1):30-44.

8. Brookfield S, Dean J, Forrest C, Jones J, Fitzgerald L. Barriers to Accessing Sexual Health Services for Transgender and Male Sex Workers: A Systematic Qualitative Meta-summary. AIDS Behav. 2020;24(3):682-96.

9. Brown C, Roucoux G, Dimi S, Fahmi S, Jeevan RB, Chassany O, et al. Evaluating Clinician Expectations of mHealth Solutions to Increase Rapid-Screening for HIV and Hepatitis in Migrant Populations in France: Qualitative Study. JMIR Hum Factors. 2023;10:e41861.

10. Burchell AN, Lisk R, Yeung A, Rana J, Bacon J, Brunetta J, et al. Community-Directed Bacterial Sexually Transmitted Infection Testing Interventions Among Men Who Have Sex With Men: Protocol for an E-Delphi Study in Toronto, Canada. JMIR Res Protoc. 2019;8(7):e13801.

11. Calin T, Green J, Hetherton J, Brook G. Disclosure of HIV among black African men and women attending a London HIV clinic. AIDS Care. 2007;19(3):385-91.

12. Cassidy C, Steenbeek A, Langille D, Martin-Misener R, Curran J. Sexual health service use among university undergraduate students in Nova Scotia. Canadian Journal of Human Sexuality. 2018;27(3):207-14.

13. Chorba T, Scholes D, Bluespruce J, Operskalski BH, Irwin K. Sexually transmitted diseases and managed care: an inquiry and review of issues affecting service delivery. Am J Med Qual. 2004;19(4):145-56.

14. Collyer A, Bourke S, Temple-Smith M. General practitioners' perspectives on promoting sexual health to young men. Aust J Gen Pract. 2018;47(6):376-81.

15. Conserve DF, Jennings L, Aguiar C, Shin G, Handler L, Maman S. Systematic review of mobile health behavioural interventions to improve uptake of HIV testing for vulnerable and key populations. J Telemed Telecare. 2017;23(2):347-59.

16. Corker E, Lorencatto F, Anderson N, Gobin M, Scott S, Michie S, et al. Acceptability and facilitators of and barriers to point-of-care HIV testing in a homeless-focused service in Gloucestershire: a qualitative evaluation. HIV Med. 2022;23(3):237-48.

17. de Munnik S, Vervoort S, Ammerlaan HSM, Kok G, den Daas C. From intention to STI prevention: An online questionnaire on barriers and facilitators for discussing sexual risk behaviour among HIV nurses. J Adv Nurs. 2017;73(12):2953-61.

18. Ekstrand M, Tydén T, Larsson M. Exposing oneself and one's partner to sexual risk-taking as perceived by young Swedish men who requested a Chlamydia test. Eur J Contracept Reprod Health Care. 2011;16(2):100-7.

19. Flowers P, Duncan B, Frankis J. Community, Responsibility and Culpability: HIV Risk-Management amongst Scottish Gay Men. J Community Appl Soc Psychol. 2000;10:285-300.

20. Flowers P, Knussen C, Duncan B. Re-appraising HIV Testing among Scottish Gay Men: The Impact of New HIV Treatments. J Health Psychol. 2001;6(6):665-78.

21. Freeman E, Howell-Jones R, Oliver I, Randall S, Ford-Young W, Beckwith P, et al. Promoting chlamydia screening with posters and leaflets in general practice--a qualitative study. BMC Public Health. 2009;9:383.

22. Gesink D, Salway T, Kimura L, Connell J. Sexual Health Knowledge, Attitudes, and Perceptions Among Men Who Have Sex With Men During Co-Occurring Sexually Transmitted Infection Epidemics in Toronto, Canada: A Qualitative Study. Sex Transm Dis. 2020;47(10):658-62.

23. Gkatzidou V, Hone K, Sutcliffe L, Gibbs J, Sadiq ST, Szczepura A, et al. User interface design for mobile-based sexual health interventions for young people: design recommendations from a qualitative study on an online Chlamydia clinical care pathway. BMC Med Inform Decis Mak. 2015;15:72.

24. Gudka S, Afuwape FE, Wong B, Yow XL, Anderson C, Clifford RM. Chlamydia screening interventions from community pharmacies: a systematic review. Sex Health. 2013;10(3):229-39.

25. Ho CL, Pan W, Taylor LD. Stigma of HIV Testing on Online HIV Forums: Self-Stigma and the Unspoken. J Psychosoc Nurs Ment Health Serv. 2017;55(12):34-43.

26. Horwitz R, Brener L, Marshall AD, Caruana T, Newman CE. Optimising community health services in Australia for populations affected by stigmatised infections: What do service users want? Health Soc Care Community. 2022;30(6):e3686-e95.

27. Kesten J, Davies C, Horwood J, May M, Gompels M, Billing A, et al. Qualitative evaluation of an education intervention for healthcare professionals on appropriate HIV testing in higher prevalence general practices in a city in the southwest of England. HIV medicine. 2017;18:65.

28. Kesten JM, Davies CF, Gompels M, Crofts M, Billing A, May MT, et al. Qualitative evaluation of a pilot educational intervention to increase primary care HIV-testing. BMC Fam Pract. 2019;20(1):74.

29. Kielly J, Kelly DV, Hughes C, Day K, Hancock S, Asghari S, et al. Adaptation of POCT for pharmacies to reduce risk and optimize access to care in HIV, the APPROACH study protocol: Examining acceptability and feasibility. Pilot and Feasibility Studies. 2018;4(1).

30. King EJ, Maman S. Structural barriers to receiving health care services for female sex workers in Russia. Qual Health Res. 2013;23(8):1079-88.

31. Kiridaran V, Chawla M, Bailey JV. Views, attitudes and experiences of South Asian women concerning sexual health services in the UK: a qualitative study. Eur J Contracept Reprod Health Care. 2022;27(5):418-23.

32. Koester KA, Fuller SM, Maiorana A, Steward WT, Zamudio-Haas S, Xavier J, et al. Implementing Multi-Level Interventions to Improve HIV Testing, Linkage-to-and Retention-in-Care Interventions. J Health Care Poor Underserved. 2016;27(3):1234-51.

33. Larcombe L, Ringaert L, Restall G, McLeod A, Hydesmith E, Favel A, et al. "Because of COVID…": The impacts of COVID-19 on First Nation people accessing the HIV cascade of care in Manitoba, Canada. PLoS One. 2023;18(8):e0288984.

34. Leblanc NM, Flores DD, Barroso J. Facilitators and Barriers to HIV Screening: A Qualitative Meta-Synthesis. Qual Health Res. 2016;26(3):294-306.

35. Leddy AM, Weiss E, Yam E, Pulerwitz J. Gender-based violence and engagement in biomedical HIV prevention, care and treatment: a scoping review. BMC Public Health. 2019;19(1):897.

36. Leidel S, Wilson S, McConigley R, Boldy D, Girdler S. Health-care providers' experiences with opt-out HIV testing: a systematic review. AIDS Care. 2015;27(12):1455-67.

37. Leitinger D, Ryan KE, Brown G, Pedrana A, Wilkinson AL, Ryan C, et al. Acceptability and HIV Prevention Benefits of a Peer-Based Model of Rapid Point of Care HIV Testing for Australian Gay, Bisexual and Other Men Who Have Sex with Men. AIDS Behav. 2018;22(1):178-89.

38. Lorch R, Hocking J, Guy R, Vaisey A, Wood A, Donovan B, et al. Do Australian general practitioners believe practice nurses can take a role in chlamydia testing? A qualitative study of attitudes and opinions. BMC infectious diseases. 2015;15(1):31.

39. Lorenc T, Marrero-Guillamón I, Llewellyn A, Aggleton P, Cooper C, Lehmann A, et al. HIV testing among men who have sex with men (MSM): systematic review of qualitative evidence. Health Educ Res. 2011;26(5):834-46.

40. Lui CW, Dean J, Mutch A, Mao L, Debattista J, Lemoire J, et al. HIV Testing in Men who have Sex with Men: A Follow-up Review of the Qualitative Literature since 2010. AIDS Behav. 2018;22(2):593-605.

41. Manirankunda L, Wallace A, Ddungu C, Nöstlinger C. Stigma Mechanisms and Outcomes among Sub-Saharan African Descendants in Belgium-Contextualizing the HIV Stigma Framework. Int J Environ Res Public Health. 2021;18(16).

42. McDonagh LK, Saunders JM, Cassell J, Bastaki H, Hartney T, Rait G. Facilitators and barriers to chlamydia testing in general practice for young people using a theoretical model (COM-B): a systematic review protocol. BMJ Open. 2017;7(3):e013588.

43. McDonagh LK, Saunders JM, Cassell J, Curtis T, Bastaki H, Hartney T, et al. Application of the COM-B model to barriers and facilitators to chlamydia testing in general practice for young people and primary care practitioners: a systematic review. Implement Sci. 2018;13(1):130.

44. Metcalfe R, Laird G, Nandwani R. Don't ask, sometimes tell. A survey of men who have sex with men sexual orientation disclosure in general practice. Int J STD AIDS. 2015;26(14):1028-34.

45. Middleton M, Somerset S, Evans C, Blake H. Test@Work Texts: Mobile Phone Messaging to Increase Awareness of HIV and HIV Testing in UK Construction Employees during the COVID-19 Pandemic. Int J Environ Res Public Health. 2020;17(21).

46. Mocelin HJS, de Jezus SV, dos Santos Almeida Negri L, Borges BJP, da Silva AI, Maciel ELN. Barriers and facilitators to confronting HIV/aids and syphilis experienced by Venezuelan women living in Brazil. Revista Panamericana de Salud Publica/Pan American Journal of Public Health. 2023;47.

47. Mullens AB, Duyker J, Brownlow C, Lemoire J, Daken K, Gow J. Point-of-care testing (POCT) for HIV/STI targeting MSM in regional Australia at community 'beat' locations. BMC Health Serv Res. 2019;19(1):93.

48. Nanhoe AC, Visser M, Omlo JJ, Watzeels A, van den Broek IV, Götz HM. A pill for the partner via the chlamydia patient? Results from a mixed method study among sexual health care providers in the Netherlands. BMC Infect Dis. 2018;18(1):243.

49. Natoli L, Guy RJ, Shephard M, Whiley D, Tabrizi SN, Ward J, et al. Public health implications of molecular point-of-care testing for chlamydia and gonorrhoea in remote primary care services in Australia: a qualitative study. BMJ Open. 2015;5(4):e006922.

50. Padovese V, Farrugia A, Almabrok Ali Ghath S, Rossoni I. Sexually transmitted infections' epidemiology and knowledge, attitude and practice survey in a set of migrants attending the sexual health clinic in Malta. J Eur Acad Dermatol Venereol. 2021;35(2):509-16.

51. Paiva V, Segurado AC, Filipe EM. Self-disclosure of HIV diagnosis to sexual partners by heterosexual and bisexual men: a challenge for HIV/AIDS care and prevention. Cad Saude Publica. 2011;27(9):1699-710.

52. Palinkas LA, Robertson AM, Syvertsen JL, Hernandez DO, Ulibarri MD, Rangel MG, et al. Client perspectives on design and implementation of a couples-based intervention to reduce sexual and drug risk behaviors among female sex workers and their noncommercial partners in Tijuana and Ciudad Juárez, México. AIDS Behav. 2014;18(3):583-94.

53. Patterson TL, Semple SJ, Chavarin CV, Mendoza DV, Santos LE, Chaffin M, et al. Implementation of an efficacious intervention for high risk women in Mexico: protocol for a multi-site randomized trial with a parallel study of organizational factors. Implement Sci. 2012;7:105.

54. Ransom JE, Siler B, Peters RM, Maurer MJ. Worry: women's experience of HIV testing. Qual Health Res. 2005;15(3):382-93.

55. Reed JL, Huppert JS, Gillespie GL, Taylor RG, Holland CK, Alessandrini EA, et al. Adolescent patient preferences surrounding partner notification and treatment for sexually transmitted infections. Acad Emerg Med. 2015;22(1):61-6.

56. Reed JL, Punches BE, Taylor RG, Macaluso M, Alessandrini EA, Kahn JA. A Qualitative Analysis of Adolescent and Caregiver Acceptability of Universally Offered Gonorrhea and Chlamydia Screening in the Pediatric Emergency Department. Ann Emerg Med. 2017;70(6):787-96.e2.

57. Ricketts EJ, Francischetto EO, Wallace LM, Hogan A, McNulty CA. Tools to overcome potential barriers to chlamydia screening in general practice: qualitative evaluation of the implementation of a complex intervention. BMC family practice. 2016;17:33.

58. Ruffinen CZ, Sabidó M, Díaz-Bermúdez XP, Lacerda M, Mabey D, Peeling RW, et al. Point-of-care screening for syphilis and HIV in the borderlands: challenges in implementation in the Brazilian Amazon. BMC Health Serv Res. 2015;15:495.

59. Schäffer D, Rensmann W, Michel S. Community-based rapid HIV testing for drug users in low-threshold service - TEST IT. Suchtmedizin in Forschung und Praxis. 2013;15(1):13-7.

60. Seguin M, Dodds C, Mugweni E, McDaid L, Flowers P, Wayal S, et al. Self-sampling kits to increase HIV testing among black Africans in the UK: the HAUS mixed-methods study. Health Technol Assess. 2018;22(22):1-158.

61. Snelgrove JW, Jasudavisius AM, Rowe BW, Head EM, Bauer GR. "Completely out-at-sea" with "two-gender medicine": a qualitative analysis of physician-side barriers to providing healthcare for transgender patients. BMC Health Serv Res. 2012;12:110.

62. Spicer N, Bogdan D, Brugha R, Harmer A, Murzalieva G, Semigina T. 'It's risky to walk in the city with syringes': Understanding access to HIV/AIDS services for injecting drug users in the former Soviet Union countries of Ukraine and Kyrgyzstan. Globalization and Health. 2011;7.

63. Theunissen KA, Schipper P, Hoebe CJ, Crutzen R, Kok G, Dukers-Muijrers NH. Barriers to and facilitators of partner notification for chlamydia trachomatis among health care professionals. BMC Health Serv Res. 2014;14:647.

64. Vermandere H, Aguilera-Mijares S, Martínez-Vargas L, Colchero MA, Bautista-Arredondo S. Developing HIV assisted partner notification services tailored to Mexican key populations: a qualitative approach. BMC Public Health. 2021;21(1):555.

65. Williams JR, Gonzalez-Guarda RM, Ilias V. Trauma-Informed Decision-Making Among Providers and Victims of Intimate Partner Violence During HIV Testing: A Qualitative Study. J Assoc Nurses AIDS Care. 2017;28(5):819-31.

66. Witzel TC, Rodger AJ, Burns FM, Rhodes T, Weatherburn P. HIV Self-Testing among Men Who Have Sex with Men (MSM) in the UK: A Qualitative Study of Barriers and Facilitators, Intervention Preferences and Perceived Impacts. PLoS One. 2016;11(9):e0162713.

67. Witzel TC, Weatherburn P, Rodger AJ, Bourne AH, Burns FM. Risk, reassurance and routine: a qualitative study of narrative understandings of the potential for HIV self-testing among men who have sex with men in England. BMC Public Health. 2017;17(1):491.

68. Wood H, Hall C, Ioppolo E, Ioppolo R, Scacchia E, Clifford R, et al. Barriers and facilitators of partner treatment of chlamydia: A qualitative investigation with prescribers and community pharmacists. Pharmacy. 2018;6(1).

69. Worthington C, Myers T. Factors underlying anxiety in HIV testing: risk perceptions, stigma, and the patient-provider power dynamic. Qual Health Res. 2003;13(5):636-55.

70. Wright T, Nicholls EJ, Rodger AJ, Burns FM, Weatherburn P, Pebody R, et al. Accessing and utilising gender-affirming healthcare in England and Wales: trans and non-binary people's accounts of navigating gender identity clinics. BMC Health Serv Res. 2021;21(1):609.

71. Xu W, Liang P, Wang C. Facilitators and Barriers for Chlamydia and Gonorrhea Testing in Female Sex Workers: A Scoping Review. Open Forum Infect Dis. 2023;10(8):ofad397.

72. Yeung A, Temple-Smith M, Fairley C, Hocking J. Narrative review of the barriers and facilitators to chlamydia testing in general practice. Aust J Prim Health. 2015;21(2):139-47.

73. Young J, Ablona A, Klassen BJ, Higgins R, Kim J, Lavoie S, et al. Implementing community-based Dried Blood Spot (DBS) testing for HIV and hepatitis C: a qualitative analysis of key facilitators and ongoing challenges. BMC Public Health. 2022;22(1):1085.

74. Zapata JP, Petroll AE, Quinn KG, Zamantakis A, John SA. Implementation determinants of HIV Self-Testing among young sexual minority men. Arch Public Health. 2023;81(1):113.
