# Supplementary material for: Barriers and facilitators to the diagnosis of HIV and other STIs in primary care within publicly funded healthcare systems: A systematic review of qualitative studies
Source: PLoS One. 2026 Feb 5;21(2):e0341919. doi: 10.1371/journal.pone.0341919 (PMC12875586; doi:10.1371/journal.pone.0341919)
Supplement: S4 File — (DOCX) [file pone.0341919.s004.docx]

**Table S4_1. Results of individual studies**

| # | AUTHOR, year | BARRIERS:  patient level | BARRIERS:  provider level | BARRIERS:  system level | FACILITATOR:  Patient level | FACILITATOR:  Provider level | FACILITATOR:  system level |
| --- | --- | --- | --- | --- | --- | --- | --- |
| 1 | **Adams_2020 (1)** | - Shame (being gay) - Stigma (being gay) - Talk about sex and sexual health | - HIV health promotion is too explicit and too focused on sexual practices | - Inaccessible resources - Lack of access to quality information - HIV health promotion is too explicit and focused on sexual practices - Access to health care is expensive and time-consuming |  |  |  |
| 2 | **Adedimeji_2015 (2)** | - Cost - Delay in accessing services - Discriminatory policies - Ignorance - Lack of support from religious leaders/social networks - Language limitations - Location/organization of HIV testing - Negative attitudes - Poor provider–patient relationships - Stigma | - Poor provider–patient relationships - Stigmatizing attitudes | - Cost - Unavailability of interpreter services - Lack of support from religious leaders/social networks - Delay in accessing services - Discriminatory policies (stereotype of Africans as HIV carriers) |  |  | Promotion of testing in pharmacies, schools and colleges |
| 3 | **Ahmaro_2021 (3)** | - Ignorance of complications if untreated - Unsure of symptoms - Worry (negative comments peers) | - Low awareness of pharmacy testing service | - Pharmacies are crowded places | - Pharmacies are convenient | - Counselled of pharmacist | - Need of private area in pharmacy - Pharmacy is convenient for patients |
| 4 | **Ahmaro_2022 (4)** | - Young people do not accept the test - Young people do not request the test |  | - Need of training on communicating with young people - Pharmacist snot want jeopardize relationship with client |  |  | - Test and treatment - Offer testing to all in ECP consultation |
| 5 | **Aicken_2016 (5)** | - Anxiety - Conceal sexual healthcare use - Embarrassment (sexual history) |  |  | - Providing faceless information - Perceived legitimacy - Self-testing | - Personal support in positive results | - Telephone helpline for human support - Confidentiality and data security - Quicker and easier access to STI testing with smartphone |
| 6 | **Åkerman_2017 (6)** | - Lack of knowledge of healthcare system - Language difficulties - Not perceive at risk of HIV |  |  | - Key role of partner: interpreter, support - Positive attitude on HIV prevention |  | - Opportunity of HIV testing - Information about where to go be HIV testing |
| 7 | **Allison_2017 (7)** |  | - Lack of time - Forgetting to offer the 3C in non-sexual health consultation | - Not enough kits of Chlamydia - Lack of privacy in reception area | - Financial incentives |  | - Educational workshops |
| 8 | **Apers_2020 (8)** |  | - Personal discomfort - Fear of offending patients - Difficulties to identify patient´s sexual orientation - Lack of guidelines - Language problems - Limited knowledge on benefits of early HIV-diagnosis - Misconceptions about HIV risk - Time constraints | - Incompatibility of HIV testing with family consultation - Time constrains |  | - Good patient-physician relationship - Standard questions on sexual health | - Official screening recommendations - Personal training of GP |
| 9 | **Balfe_2009 (9)** | - Anxiety about social consequences - Be judged by their GP - Feelings of invulnerability - Shame and embarrassment - Stigma |  | - Cost of STI testing | - Required for a employer - Unprotected sex with casual partner - Symptoms of infection - Knowing someone with a STI - Transitional moment - Damage for fertility - Protection of partners - Testing is a responsible practice |  | - Government promotion campaign - Media promotion campaign |
| 10 | **Balfe_2010 (10)** | - Identity (of "good girl") threat - Staff can know family or friends |  | - Screening from paraprofessionals | - Concerns about reproductive health - Concerns about previous risky activities | - Health professionals consider screening a normal practice - Screening by women | - Notification of results by phone |
| 11 | **Bedert_2021 (11)** | - Fear of consequences of HIV - Lack of symptoms - No GP acceptance of their sexual identity - No GP acceptance of their sexual practice - Perceived low risk of HIV | - Problematic communication - Misclassified HIV-related symptoms - Misclassified for AIDS defining illnesses | - Limited Health system capacity - Long waiting times |  |  |  |
| 12 | **Bilardi_2013 (12)** | - Cost - Doubts on accuracy/ reliability of the test - Lack of professional support in event of HIV+ - Long waiting times - Perceived to be a low risk - Rapid HIV tests no check for other STI’s |  |  | - Quick, ease to use and convenient - Eliminate waiting times to see a clinician and return for results - Privacy and discretion - Less resource intensive thereby reducing demands on health services - Painless - Reduce stress and anxiety - Especially useful in rural areas - Most men still considered blood tests to be the ‘gold standard’ in testing |  |  |
| 13 | **Blondell_2021 (13)** | - Lack of privacy - Unknown HIV risks |  |  | - Privacy (PITC; HIVRT; HIVST) - Cost (PITC; HIVST) - Relation with healthcare provider (PITC) - Accuracy (HIVRT) - Convenience (HIVRT; HIVST) - Risk perception (HIVRT) - Symptoms (PITC) - Technical and emotional support (HIVST) | - Healthcare provider–patient relationship (PITC) - Patient counselling (PITC) - Greater expertise and professionalism | - HIVRT in medical clinic |
| 14 | **Boyce_2012 (14)** | - Denied services in the public STI clinic - Fear of discrimination  (sexual identity and behaviour) - Fear of STI/HIV-related stigma/discrimination - Fear of testing - Lack of social support - Long waits - Mistreatment (transphobic attitude) | Lack of professional support available in the event of HIV+ |  | Peer support  Anonymity and discretion |  | - Free service - Specialized clinics (more comfortable for transgender) - Convenient location |
| 15 | **Boydell_2017 (15)** | - Anxiety - Bad communication/support around HIV - Social norms for no regular testing - Stigma |  | Difficulties in accessing sexual health services | - Support and encouragement from friends - Personal responsibility - Exposure to HIV risk (unprotected sex) - Open communication sexual health - Intimate relationship - Trust and friendship with partners |  |  |
| 16 | **Brendstrup_1990 (16)** | - Bisexual men are not at risk for AIDS - Not adequate treatment now - Psychological crisis with positive results |  |  |  |  |  |
| 17 | **Brugha_2011 (17)** |  |  | - Screening offered by pharmacist - Payment required for test - Screening by a receptionist or non-healthcare professional - Women: screening offered by man |  |  | - Screening by doctor and nurses - Self-testing at home - Settings for screening: general practice, student health services, specialist STI services and family planning clinics - Women: screening by female professional |
| 18 | **Christianson_2010 (18)** |  | - Unclear information of results - Bad communication | - Difficulty getting an appointment - Resistance against testing by the staff | Easier for women (regular visits) | - Friendly professional - Short pretest conversation | - Quick and easy testing - Result given by phone |
| 19 | **Day_2011 (19)** | - Appointment waiting period - Being judged and lectured - Don’t remember an appointment - Fear of the unknown - Hostile receptionists - Negative experiences - Not wanting to say you are a drug user - Shame and embarrassment | Hostile receptionists |  | Emotional preparation for the visit | Referral to other health services | - Quick access or no appointment needed - Appointment reminders - Close geographical location |
| 20 | **Denison_2017 (20)** | - Being too busy - Fear of invasive procedure - Genital examination - Perception STIs as not serious - Stigma - Underestimating risk |  | - Separate location for a blood draw - Difficulty booking an appointment - Financial cost of STI test | - Self-swabs (female) - Self-taken urine test (men) | Same sex health professional | University clinic (not meeting family or friends) |
| 21 | **Dowson_2012 (21)** | - Fear of illness/death from AIDS - Perceived low risk of HIV infection - Stigma | No explanation of the importance of testing by GP offering the test | Testing in sexual health clinic |  | More proactive approach to HIV testing | - Testing in GP's surgery - Peer education from men VIH+ |
| 22 | **Etowa_2022 (22)** | - Confidentiality - Fear of deportation - Health beliefs (not use preventive services) - HIV perceived as a death sentence - Lack of information and location - Stigma - Time commitment | - GP never discussed PEP or PrEP with patients - Lack of cultural competency - Lack of HIV awareness of GP - Racism - Judgements of health care providers | Resources limited | Self-testing kit | In-depth discussions about HIV | - Collaboration between ACB community agencies - Increase the availability of HIV information - Targeted and culturally specific messages - Community-wide educational effort - Messages to promote HIV testing - Best place for information |
| 23 | **Evans_2016 (23)** | - Cultural/religious norms - Discrimination - Fear around confidentiality - Fear of social stigma - Lack of knowledge of testing - Lack of knowledge of VIH - Lack of trust in providers - Language and cultural differences | Competence of the provider | - Inflexible working hours - Lack of trust in health system - Waiting time - Hard access to GP | - Test messages from trusted source - Possibility of getting treatment | Sincerity and trust | - Messages on other health topics - Raising awareness of VIH - Community outreach activities - Community leaders as role models - Campaign about individual’s responsibility |
| 24 | **Ewert_2016 (24)** | - Embarrassment - Image of masculinity - Lack of concern - Not knowing where to go - Privacy - Trust |  | - Cost (international student) - Not knowing where to go |  | GP initiated discussion of sexual health | Promoting sexual health in schools, TV, facebook, etc… |
| 25 | **Fernández-Gerlinger**  **_2013 (25)** | - Cost of public health - Embarrassment - Fear of illness/results - Lack of information about screening methods - Protection due to living in France - Questioning marital fidelity - Religion - Think: HIV only with sexual relations |  | - Information overload - Cost of public health - Lack of information about screening methods |  |  |  |
| 26 | **Figueira_2022 (26)** | - Recognition of users by others - Stigma | - Difficulty in collecting adequate blood sample - No psychological training to provide results | - Complexity of referral process - Lack of information on continuum of care - Need to be at least 18 years old to test | - Reduced waiting time - Privacy and confidence - Change of sexual partner - Unprotected sex - Never been tested | - Confidence and competence - Expertise and technical skills for testing - Training for testing | - Proximity - Shorter waiting time - Availability at weekends - Private area (confidentiality) - Training for testing |
| 27 | **Fleming_2020 (27)** | - Embarrassment and stigma - Influence of peers - Lack of knowledge of STIs - Perceptions of invulnerability to STIs - Testing as surveillance on their behaviour |  |  | - Knowledge of the risk of STIs - Influence of peers in facilitating testing |  | - Non-medical setting for testing - Access to testing at college - Incentives (not necessary money) - Publicity and reminders - Simultaneous prior education with testing |
| 28 | **Flowers_2017 (28)** | - Low perceived accuracy of the kit - Negative consequences of reactive test results - Poor trust in the kit (self-test) - Skills to use the kit correctly | - Lost opportunities for engagement with services/staff - Deracinating HIV from an holistic health |  | - Self-test avoid stigma - Self-test: Discretion and privacy - Self-test: Convenience and immediacy - Self-test: Convenience accessing test result |  | - Reach vulnerable populations - Rationalize clinical time - Rationalize resources |
| 29 | **Godin_2000 (29)** | - Anxiety and fear - Disclose of sexual identity/unsafe practices - Fear of positive test result - Fear of ridicule - Need to face reality - Need to find a understanding doctor - Physicians´ negative attitudes - Prejudiced GP against homosexual men | Physicians´ negative attitudes |  | - Prompt reassurance to eliminate anxiety - Role of partners and close friends |  |  |
| 30 | **Grandahl_2020 (30)** | - Detection of only two infections - Handling personal data - Lack of counseling - Language - Uncertainty about procedure |  |  | - Avoiding clinical visits - Convenience - Ease to access and use - Free test - Confidentiality - Rapid test result |  | - Accessibility - Change perception that free test is not a substitute of condom - Benefits for young and migrants |
| 31 | **Heijman_2017 (31)** | - Aversion to STI testing site - Burdensome testing procedures - False belief of been checking for all STIs in HIV care centres - Lack of specific knowledge - Protected by condoms | False belief of been checking for all STIs in HIV care centres |  | - Feeling vulnerable for infections - Health consciousness - Protecting partner from STI |  |  |
| 32 | **Heritage_2008 (32)** | - Concerns about receptionists - Partner tracing | - GP's professionalism - Limited time for consultations | - Rights to confidentiality - Lack of privacy on reception | Presence of their parents | Young GP for introduction and distribution of QCT kit | Urine test prefered |
| 33 | **Hocking_2008 (33)** | - Lack of knowledge about chlamydia - Lack of support for following-up partners - Lack of support for partner notification - Patient embarrassment - Religion or ethnicity of patients - Religious and cultural issues | - Bad communication with patients - Some providers prefer swabs rather than urine - Lack of knowledge about chlamydia of GP - Chlamydia screening is a difficult topic - Discomfort talking about sexual health matters | - Cost of testing - GP workload - Lack of support for partner notification - Time constraints |  | - Education of GPs - Incentive payment | - Destigmatization of chlamydia - Recall/reminder system - Education of GPs and practice staff - National chlamydia screening program - Broad community education |
| 34 | **Hogan_2010 (34)** | - Embarrassment - Fears of judgement by staff and fear of parents - Forgetting to return the sample for home testing kits - Lack of knowledge - Scared of results and outcome | Time constraints | Specific sessions for young people (worried about seeing people they knew) | - Raising awareness - More comfortable with a doctor they know - Preferences of testing at GP practice vs home | - Characteristics of doctor or nurse - Being non-judgmental |  |
| 35 | **Jones_2017 (35)** | - Embarrassment - Lack of time (less quality of consultation) - Offence due to religious matters - Unease around testing |  |  | - Trust in GP staff - Confidentiality | - Test offer in non-judgmental way - Reassuring confidentiality - Professionalism of GP to discuss sexual health | GP: raise awareness of SHS |
| 36 | **Joore_2017 (36)** | - Cost of the test - IC test: fear - Judging patients’ sexual behaviour - Stigma | - Time for discussing an HIV test - Judging patients’ sexual behaviour - Offering tests to new patients is inappropriate - Not know when to repeat screening (window period) - IC-test: a lot of IC; not applicable in primary care | - Cost of the test - IC-test: need to test too many people |  | - Clear link with immunodeficiency - General health checks may include HIV test - Collect information about sexual behaviour |  |
| 37 | **King_2017 (37)** | - Afraid of needles - Distance to the testing services - Don't know where to test - Doubt confidentiality - Nervous or fear of learning result - Never think about testing - No perceived risk - Not important for the person - Results take too long - Time away from work - Too expensive - Worried about partner´s reaction - Worried people will think I am sick |  | Too expensive | - Could get treatment if positive - To obtaining documents - Concern about one´s health - Important for job - Reassurance - Want to stop worrying - Personal motivation - Wanting to protect others |  |  |
| 38 | **Krabbenborg_2021 (38)** |  | GP unprepared for communicating HIV-positive result | Referral-to-care on weekend days | - Discreet environment - Confidentiality and anonymity - Rapid result - No cost - Accessibility of checkpoint - Absence of waiting lists - Trust between lay providers-patients | - Trust between lay providers-patients - Training to improve STI knowledge - Listening to the patient | - Absence of waiting lists - Accessibility of checkpoint - Check-in successfully linked to care - Discreet environment - More awareness about HIV testing (social media) - No cost - Quality of test procedures - Training to improve STI knowledge |
| 39 | **Lorch_2015 (39)** | Confidentiality in small town | - Time and workload constraints - GP attitude towards testing by PN | Legislation of ordering the pathology | - Increased accessibility to testing - Patients’ empowerment - Preference for PN to GP (females) | Training for PN | - Ease workload for GP - Education and training for PN - Expansion of nurse role |
| 40 | **Lorimer_2009 (40)** | - Embarrassment - Increased stigma (women) - Low risk (monogamous relationships) |  |  | - Convenience - Ease of test in urine (men) - Raised awareness | Non-medical screening setting | Less formal setting |
| 41 | **Lorimer_2013 (41)** |  |  |  | - Internet-based screening: Privacy and confidentiality - Internet-based screening: Easy and convenience |  | - Design of the Website - Invitation letter - Nearby clinic - Offer privacy and confidentiality - Website with credible and clear content |
| 42 | **Lorimer_2014 (42)** | - Gender issues (CT=promiscuous women) - Men low attendance | Reluctance to talk on sexual health with men | Lack of campaign of normalization of testing | - Convenience - Ease of access - Anonymity - Women: opportunistic screening |  | - Proactive, Internet-based screening - Confidentiality |
| 43 | **Malta_2007 (43)** | - Fears or embarrassment - Having to face the diagnostic - Stigma and discrimination | - No dialogue with GP on STI prevention - Lack of information about STI transmission - Lack of counsel and support | Lack of specialized training of GP | - Good social network - To have symptoms | Clear information on diagnosis |  |
| 44 | **Manirankunda_2009 (44)** | - Employment difficulties - Fear of death - Fear of stigma and social rejection - Financial cost - HIV positive = deportation - Lack of preventive culture - Low self-perceived risk - No information about HIV transmission - Strict treatment regimen |  | - Financial cost - Lack of transparency of screening methods |  | Not question the advice of GP to do a test. | Free treatment |
| 45 | **Manirankunda_2012 (45)** | - Migration-related barriers - Reliance on social benefits | - Lack of information on HIV among SAM - Migration-related barriers - Fear of stigmatizing patients - Unethical for undocumented patients - Cultural differences (infidelity) - Lack of culture-sensitive sexual counseling - Lack of time - Language barriers - Questionable relevance of pre-test counseling | - HIV/AIDS exceptionalism - Language barriers - Lack of time - Unethical for undocumented patients |  | - Trusting provider-patient relationship - Pre-test counselling - Training providers | - Training providers - Supporting policies development |
| 46 | **Masaro_2012 (46)** | - Confidentiality - Youths’ lack of responsibility | - Deficient in knowledge and skills - Workload - Confidentiality - Resignation and frustration over things that cannot be changed - Follow up on positive results - Status quo | - Workload - Lack of resources - Enough physical space - Lack of effectiveness of STI´screening |  | - No need to present PHN or ID - Additional follow-up care. | - Eliminating fee-for-service billing - Strategies for innovations in practice |
| 47 | **McDonagh_2020 (47)** | - Embarrassment - Fear - Guilt - Lack of knowledge - Perceived low risk - Physical skills (vulvovaginal swabs) - Stigma - Testing is not a priority - Uncomfortable disclosing sexual orientation | Time constraints | - Time constraints - Location of toilet - Registration - Lacks urgency - Strained system - Getting appointments | - Credible sources - Friend referrals - Example of the technique | - Guidelines offered in all consultations - Young person’s health check - Positive reinforcement - GP offering testing - More information and awareness - Moral obligation to others | - Challenge perceptions of chlamydia - Flexible appointments - Alternative sampling methods - Target younger ages groups - Use imagery or data to alter beliefs - Increase information and awareness - Communication on chlamydia - School-based education - Reminder letter - Alternative staff for screening - Clear instructions for self-sampling kits - Home self-sampling - Offer guidelines - Online testing via GP website |
| 48 | **McNulty_2004 (48)** |  | - Lack of knowledge - Negative attitude to screening - Need for contact tracing. - No confidence on what information should be giving to the patient - Lack of time - Upset doctor–patient relationship - Reluctant to discuss sexual health - Resistance to discuss screening at cervical smear examinations | - Staffing - Finances - Time for an appointment | Clear lines of responsibility for contact tracing | - Skills to discuss sexual health - Test at cervical smear consultation | - Education of staff and patients - Non-invasive specimens - Patient leaflets pre-appointment - Properly resourced program - Specific clinics for testing - Trained staff in GP |
| 49 | **McNulty_2010 (49)** |  | - Not convinced of the benefits of screening - Not comfortable raising screening - Unsure of the public health benefit - Screening more appropriate in specific clinics - Time pressure - Awareness of screening for men - Difficult in the presence of parents - Insufficient training - Opportunistic screens might cause offence | - Insufficient training - Interested person not supported by the team - Confidentiality issues in reception - Screening kits not readily accessible |  | Computer templates at consultations | - Computer reminders/alerts - Easy availability of screening kits - Posters and leaflets - To become officially a priority - Training |
| 50 | **Mills_2006 (50)** | - Anxiety - Discomfort - Stigma (women) |  | Uneasy vaginal swab | - Implications for sexual relationships - Happy with screening |  |  |
| 51 | **Mitra 2006 (51)** | - Concerns about talking about HIV-STI - Cross-cultural communication barriers - Discrimination - Fear of being judged - Fear of disclosure when accessing care - Lack of confidentiality - Lack of knowledge about ART - Lack of knowledge about testing - Lack of knowledge on transmission - Lack of relationship with provider - Lack of use of screening services - Language limitations of the patient - Loss of social support - Poor continuity of care - Stigma associated with testing | - Issues for multicultural populations - Poor continuity of care - Conceptual issues for multicultural populations - Lack of cultural sensitivity (practitioners) - Time restraints in family practice | - Lack of funding for organizations working with immigrants - Poorer post-test follow-up (anonymous testing) - lack of partner notification - Lack of resources or funding for immigrant health services - Lack of language services - Ineffective VCT strategies - Health care access challenges - Lack of access to health services | - Culturally accepted strategies - Control over consequences of testing - Cultural support community - Enhanced client confidentiality (anonymous testing) - Confidentiality | - Confidentiality - Appropriate language services at the point of care - Better counseling | - Scope for longer consultation - Improved access to PC - Increased language services - Multidisciplinary care environment - Adaptation to needs of target group - Cultural sensitivity of providers - Integrated HIV services - Increased HIV education |
| 52 | **Navaza_2012 (52)** | - Blood as symbol of life - Conspiracy beliefs - Cultural difference. - Fear of being repatriated - Fear of positive results - Lack of information about Spanish health system - Lack of preventive medicine in countries of origin - Linguistic barriers - Misconceptions about HIV - Not much reliance on health care professionals - Problems to know results due to mobilization to another community - Religious factor. - Stigmatized in case of HIV+ |  |  |  |  |  |
| 53 | **Normansell_2016 (53)** | - Difficulty with access - Embarrassment and stigma - Fear of positive results - GP judgmental - Insufficient expertise of GP - Long wait to be seen - Postal kit: risk of being opened by a family member | - Insufficient expertise of GP - Cultural norms | - Inaccessible information - Difficulty with access - Long wait to be seen | - Convenience of a postal sample kit - Confidential testing - Perceived value of testing - Reassurance of partner status - Rapid testing - Free treatment available in clinics | - Personalized information - Expert care | - Sexual education in the school - Free treatment available in the clinics - Rapid testing in clinic - Expert care. - Competent service to testing |
| 54 | **Oliver de Visser_2013 (54)** | - Assumption that monogamy is protective - Lack of knowledge of how to test - Low perceived risk - Shame and embarrassment - Stigma |  |  | - Testing more accessible - Normalization of testing - Testing as normative (i.e. not unusual) | - Medical professionals presented as nonjudgmental | Testing more accessible |
| 55 | **Peters_2022 (55)** | - Fear of judgement - Fear of needles - Fear of possible costs of STI test - Fear of results STI test - Lack of awareness of confidentiality - Lack of identification as sex worker - Lack of STI knowledge - Lack of trust in STI clinic - Low STI risk perception - Negative attitude of STI clinic - Negative social norms towards MSM-MSW - Shame for sexuality - Stigma (MSM and MSW) |  | Low awareness of SHS STI clinic | - No stigma - Identifying as sex worker - Social responsibility (no transmission to sex partners) - Understanding benefits STI test - Trust in STI clinic - High STI risk perception - Awareness of confidentiality | Informal and positive communication | - MSW-MSM peer group meetings - Promotion of MSW-MSM SHS and IFW - Providing STI online information - Providing self-sampling STI kits - Using non-stigmatizing communication messages - Providing social support in consultations |
| 56 | **Prost_2007_a (56)** | - Confidentiality and privacy. - Impact of alcohol and drugs in testing - Safety issues - Social venues inappropriate for testing - Stigmatizing impact of HIV |  |  |  |  | - Alternative models for MSM ("health bus") - Information in homosexual venue |
| 57 | **Prost_2007_b (57)** | - Anxiety about confidentiality - Fear of HIV stigma in African community - Potential lack of professionalism | - Potential lack of professionalism | Care pathways: follow-up | - Support to newly diagnosed - Rapid result - Fast referral to HIV services of positives | Fast referral to HIV services of positives | - Community mobilization - Sensitization in churches, schools and African social venues - Alternative methods (mobile VCT) - Efficient referrals to sexual health services - Local appropriate testing algorithm - Offer other general health checks along with HIV test - Support to the newly diagnosed - Training guidelines for VCT counsellors |
| 58 | **Rana_2022 (58)** | - Compromised anonymity and social stigma - HIV non-disclosure criminalization - Lack of anonymity (waiting room) - Lack of clarity on use of case and partner information - Language - Long distance to clinic - Long waiting time - Services separated by gender (confusion in transgenders) - Short opening hours of clinic - Stigma - Stigmatizing language - Uncomfortable clinic ambience - Undocumented persons (fear to deportation) | - Making assumptions about who requires a test based on demographics - Discrimination on gender issues - Lack of consistency and clarity on testing | - Long waiting time - Uncomfortable clinic ambience - Few clinics inclusive to all men - Long distance to clinics - Short opening hours of clinic - Clinics not welcoming ethno-racial minorities - Stigmatizing language | - Confidential testing - Results online - Short time in the waiting room - Clear communication of follow-up steps - Self-collecting samples - Having symptoms of risky sexual encounters - Taking Pr-EP for HIV prevention - Clarify how test information is utilized by public health | - Destigmatizing informed care - Inform what to do in case of positive test - Normalize sexual activity questions - Information on follow-up steps for testing - Information on tests needed by sexual exposures - Professional and non-judgmental interactions - Information of how results will be made available | - Posters with transgender language - Waiting room: call system by numbers no names - More sexual clinics inclusive to all men - Consistence in sexual history intake - Accessible by transit - Use of patient-centered language - Evening and weekend hours - Destigmatizing informed care - Results online - Posters with information in multiple languages |
| 59 | **Reisen_2014 (59)** | - Bureaucracy of risk assessment - Embarrassment - Fear of being positive - Fear of lack of confidentiality - Fear of needles - Lack of correct information - Lack of money - Lack of support - Stigma - Time spent waiting to be seen - To be unaware of their right to be tested | Need of several appointments | - Co-payments in some procedures - Information on the right to be tested - Cost of transport - Bureaucracy of risk assessment | - Sense of responsibility - Quick test - Risk perception - Support from friends and partners |  | Test in non-profit organizations (easier, faster and more comfortable) |
| 60 | **Scheim_2017 (60)** | - Difficulties accessing sexual healthcare - Disclosure of trans status and discussion of genital status - Fear of positive results - Few trans-friendly health resources - Lack of provider knowledge - Medical mistrust - Refusing care and testing - Uncomfortable clinic experience - Uncomfortable with the binary division of services | - Lack of provider knowledge - Provider perceptions of low risk - Inappropriate language | - Discrepancy between trans-inclusive policy and practice - Limited clinic capacity to meet STI testing needs | - Sense of responsibility to partners - Testing as part of a general healthcare routine - Recent HIV risk behavior - Access to trusted provider |  | - Low-barrier services - Integration of testing with gender-affirming treatment |
| 61 | **Seedat_2014 (61)** | - Cost and eligibility - Discrimination and stigma - Fear of disease status - Isolation - Lack of awareness and knowledge of diseases - Lack of confidence - Lack of confidentiality - Lack of knowledge (screening services or health-system) - Low perception of risk - Low priority on immigrants - Misunderstanding about health system - Poorer health-seeking behaviour (men) | - Cultural insensitivity - Inhospitality - Discrimination and stigma | - Lack of advocacy and promotion - Lack of confidentiality - Lack of psycho-social support services - Low awareness of health professionals - Poor link with community organizations - Capacity/funding shortages for community organizations - Migrant unfriendly services | Raise awareness of diseases and screening in communities | Ensure confidentiality | - Build migrants confidence to access health services - Stronger collaboration with community organizations - Engage faith-based organizations - Ensure confidentiality - Improve hospitality and sensitivity to migrant health needs - Increasing language support - Better access features - Increase psycho-social support - Package of care for migrants - Outreach for isolated migrants - Appropriate promotion of screening - Raise awareness of diseases and screening in communities |
| 62 | **Shangase_2015 (62)** | - Cultural barriers (gender of GP) - Discrimination because of ethnicity - Lack of Information on available HIV services - Lack of Information of procedures - Language - Price of condoms - Traditional medicine | - Lack of Information about available HIV Services - Communication with users - Lack of Information about procedures | - Lack of black community representation - Lack of Information about available HIV Services |  |  |  |
| 63 | **Shoveller_2009 (63)** | - Concern about confidentiality (rural areas) - Concern about privacy - Disclosing risky sexual behaviour to clinicians - Feminized decor of the clinic - Homophobia and heterosexism - Hours of operation of the clinic - Lack of knowledge of testing methods (urine sample) - Poor access to public transportation - Stress (clinic waiting/reception areas) - Women: erroneous belief that Pap smears include STI testing | - Confidentiality of GP - Homophobia and heterosexism - Erroneous belief that the PAP includes STI testing | - Poor access to public transportation - Limited-service hours - Clinics are ‘feminised’ Social spaces spaces because of decor - Knowledge gaps (for example, about Pap tests, etc…) |  |  | - More ‘‘youth friendly’’ clinics - More gender-neutral clinic decor |
| 64 | **Somerset_2021 (64)** | - Being in a male-dominated industry (construction) - Difficulty accessing GP services - Fear of needle stick injury - Inability to book medical appointments in advance - Lack of awareness of alternative locations to testing - Lack of support for health-seeking at work - Potential loss of pay by attending checks - Reluctance to seek health assistance until they were symptomatic - Stereotypical views about risk - Trouble getting time off |  | Lack of awareness of alternative locations to access testing | - Quick results - Convenience of having a health check at work - Perception of HIV risk - Offers peace of mind - Ease of access of testing |  | Convenience of location |
| 65 | **Thornton_2012 (65)** |  | - Few time with patient to consent appropriately - Exceptionalist of HIV testing - Not having sufficient time to test - Stigma and stereotype - Confidentiality - Misconceptions of VIH | - Target testing: perpetuate stigma - Management of results | - Routine testing: reduces stigma - Non-invasive sample (oral fluid) | - View HIV test as a public health intervention - Change of staff misperceptions - Knowing how follow positive patients - Knowledge of HIV and its treatment - Routine testing: reduces stigma - Answer patient questions | - HIV testing is cost-effective - PC: the most appropriate for routine testing - Specialist staff for positive results - Training around HIV - View HIV test as a public health intervention |
| 66 | **Uusküla_2006 (66)** | - Afraid and ashamed - Confidentiality - Long waiting for medical appointments. - Misperception of STIs as ‘‘symptomatic’’ - Poor general awareness of STI - Privacy in rural areas - Public stigmatization - STI related to sexual behaviour and immoral lifestyle. - Time constraints | Time constraints | - Time constraints - Underestimation of sexual activity of young people | - Value Health - Home sampling method - Description[s] of how to carry out the testing - Trust in the professionalism of the medical staff - Awareness that ITS can be asymptomatic | Emphasize benefits of home testing | - Campaign on benefits of home testing - Sexual education at young ages - Need of proactive education - Importance of publicity before mailing test kits - Social media: perception of STI prevention |
| 67 | **Vaughan_2010 (67)** | - The clinical setting is public (waiting rooms) - Delay for results - Difficulties talking about sexual health - Embarrassment - Lack of knowledge about STIs |  |  | - Positive impact of taking the test - Peace of mind - Anonymous - Convenience (testing packs on-campus) - Little-to-no embarrassment and shame - Convenience of text messages for negative results - Use of ‘friendly’ language | Use of ‘friendly’ language | - Use of urine test - Convenience of text messages for negative results - Presence of peer volunteers - Non-medical setting - Complete privacy |
| 68 | **Wagg_2020 (68)** | - Chlamydia infection is seen as a personal failure - Cost - Embarrassment about asking the GP - Fear of stigmatization - Geographical location - Insufficient knowledge - Perceived judgement from health professionals - Perception of risk | - Responsibility to provide information - Perceived judgement from health professionals | Not adequate STI education | - Increased knowledge reduces self-stigma - Confidence of negative result |  |  |
| 69 | **Wallace_2012 (69)** | Language barriers | - Family members present - Time within consultations - Privacy of contact with patients - Attending for no sexual health issue - Comprehension barriers - Forgetting to offer a screen - Invasion of patients’ privacy - Language barriers | - Logistic of testing in the surgery - Language barriers |  | - Attending for a sexual issue - Attending for cervical screening - Attending for contraception |  |
| 70 | **Woodbridge_2015 (70)** | Men: low frequency tester | - Men: low priority of sexual health - Difficulties perceive risk (asymptomatic STI) - Disclosure of risky behaviour - Find the right language - Lack of time in consultations - Men: Difficult sexual consultations with female GP - Questioning patients without symptoms: invasive and inappropriate | Difficulty to sexual health and medical education |  | - Gender of GP (asking about sexual matters) - Interactional delicacy | - Formal medical education online - Rational risk assessment based on epidemiology |

3C=Chlamydia screening, signposting to contraceptive services, free condoms; ACB=African, Caribbean and Black; ART=Antiretroviral treatment; GP=General practice; HIVRT=HIV rapid testing; HIVST=HIV self-testing; HNS=National Health Service; MSM=Men who have sex with men; MSM-MSW=Male sex workers who have sex with men; PHN=Personal health number; PITC=Provider-initiated HIV testing and counseling; PN=Practical nurse; PrEP=Prophylaxis pre-exposition; SAM=Sub-Saharan African migrants; SHS=Sexual healthcare services; STI=Sexually transmitted infections; TPB=Theory of planned behavior; VCT=Voluntary counselling and testing.

|  |
| --- |
|  |
|  |
|  |
|  |
|  |

**REFERENCES**

1. Adams J, Coquilla R, Montayre J, Manalastas EJ, Neville S. Views about HIV and sexual health among gay and bisexual Filipino men living in New Zealand. International Journal of Health Promotion and Education. 2021;59(6):342-53.

2. Adedimeji AA, Asibon A, O'Connor G, Carson R, Cowan E, McKinley P, et al. Increasing HIV testing among African immigrants in ireland: challenges and opportunities. J Immigr Minor Health. 2015;17(1):89-95.

3. Ahmaro L, Lindsey L, Forrest S, Whittlesea C. Young people's perceptions of accessing a community pharmacy for a chlamydia testing kit: a qualitative study based in North East England. BMJ Open. 2021;11(9):e052228.

4. Ahmaro L, Lindsey L, Forrest S, Whittlesea C. Investigating community pharmacists' perceptions of delivering chlamydia screening to young people: a qualitative study using normalisation process theory to understand professional practice. Int J Pharm Pract. 2022;30(6):507-13.

5. Aicken CR, Fuller SS, Sutcliffe LJ, Estcourt CS, Gkatzidou V, Oakeshott P, et al. Young people's perceptions of smartphone-enabled self-testing and online care for sexually transmitted infections: qualitative interview study. BMC Public Health. 2016;16(1):974.

6. Åkerman E, Essén B, Westerling R, Larsson E. Healthcare-seeking behaviour in relation to sexual and reproductive health among Thai-born women in Sweden: a qualitative study. Cult Health Sex. 2017;19(2):194-207.

7. Allison R, Lecky DM, Town K, Rugman C, Ricketts EJ, Ockendon-Powell N, et al. Exploring why a complex intervention piloted in general practices did not result in an increase in chlamydia screening and diagnosis: a qualitative evaluation using the fidelity of implementation model. BMC Fam Pract. 2017;18(1):43.

8. Apers H, Nöstlinger C, Van Beckhoven D, Deblonde J, Apers L, Verheyen K, et al. Identifying key elements to inform HIV-testing interventions for primary care in Belgium. Health Promot Int. 2020;35(2):301-11.

9. Balfe M, Brugha R. What prompts young adults in Ireland to attend health services for STI testing? BMC Public Health. 2009;9:311.

10. Balfe M, Brugha R, O'Donovan D, O'Connell E, Vaughan D. Young women's decisions to accept chlamydia screening: influences of stigma and doctor-patient interactions. BMC Public Health. 2010;10:425.

11. Bedert M, Davidovich U, de Bree G, van Bilsen W, van Sighem A, Zuilhof W, et al. Understanding Reasons for HIV Late Diagnosis: A Qualitative Study Among HIV-Positive Individuals in Amsterdam, The Netherlands. AIDS Behav. 2021;25(9):2898-906.

12. Bilardi JE, Walker S, Read T, Prestage G, Chen MY, Guy R, et al. Gay and bisexual men's views on rapid self-testing for HIV. AIDS Behav. 2013;17(6):2093-9.

13. Blondell SJ, Debattista J, Griffin MP, Durham J. 'I think they might just go to the doctor': qualitatively examining the (un)acceptability of newer HIV testing approaches among Vietnamese-born migrants in greater-Brisbane, Queensland, Australia. Sex Health. 2021;18(1):50-7.

14. Boyce S, Barrington C, Bolaños H, Arandi CG, Paz-Bailey G. Facilitating access to sexual health services for men who have sex with men and male-to-female transgender persons in Guatemala City. Cult Health Sex. 2012;14(3):313-27.

15. Boydell N, Buston K, McDaid LM. Patterns of HIV testing practices among young gay and bisexual men living in Scotland: a qualitative study. BMC Public Health. 2017;17(1):660.

16. Brendstrup E, Schmidt K. Homosexual and bisexual men's coping with the AIDS epidemic: qualitative interviews with 10 non-HIV-tested homosexual and bisexual men. Soc Sci Med. 1990;30(6):713-20.

17. Brugha R, Balfe M, Jeffares I, Conroy RM, Clarke E, Fitzgerald M, et al. Where do young adults want opportunistic chlamydia screening services to be located? J Public Health (Oxf). 2011;33(4):571-8.

18. Christianson M, Berglin B, Johansson EE. 'It should be an ordinary thing'--a qualitative study about young people's experiences of taking the HIV-test and receiving the test result. Scand J Caring Sci. 2010;24(4):678-83.

19. Day CA, Islam MM, White A, Reid SE, Hayes S, Haber PS. Development of a nurse-led primary healthcare service for injecting drug users in inner-city Sydney. Aust J Prim Health. 2011;17(1):10-5.

20. Denison HJ, Bromhead C, Grainger R, Dennison EM, Jutel A. Barriers to sexually transmitted infection testing in New Zealand: a qualitative study. Aust N Z J Public Health. 2017;41(4):432-7.

21. Dowson L, Kober C, Perry N, Fisher M, Richardson D. Why some MSM present late for HIV testing: a qualitative analysis. AIDS Care. 2012;24(2):204-9.

22. Etowa J, Tharao W, Mbuagbaw L, Baidoobonso S, Hyman I, Obiorah S, et al. Community perspectives on addressing and responding to HIV-testing, pre-exposure prophylaxis (PrEP) and post-exposure prophylaxis (PEP) among African, Caribbean and Black (ACB) people in Ontario, Canada. BMC Public Health. 2022;22(1):913.

23. Evans C, Turner K, Suggs LS, Occa A, Juma A, Blake H. Developing a mHealth intervention to promote uptake of HIV testing among African communities in the UK: a qualitative study. BMC Public Health. 2016;16:656.

24. Ewert C, Collyer A, Temple-Smith M. 'Most young men think you have to be naked in front of the GP': a qualitative study of male university students' views on barriers to sexual health. Sex Health. 2016;13(2):124-30.

25. Fernandez-Gerlinger MP, Bernard E, Saint-Lary O. What do patients think about HIV mass screening in France? A qualitative study. BMC Public Health. 2013;13:526.

26. Figueira I, Teixeira I, Rodrigues AT, Gama A, Dias S. Point-of-care HIV and hepatitis screening in community pharmacies: a quantitative and qualitative study. Int J Clin Pharm. 2022;44(5):1158-68.

27. Fleming C, Drennan VM, Kerry-Barnard S, Reid F, Adams EJ, Sadiq ST, et al. Understanding the acceptability, barriers and facilitators for chlamydia and gonorrhoea screening in technical colleges: qualitative process evaluation of the "Test n Treat" trial. BMC Public Health. 2020;20(1):1212.

28. Flowers P, Riddell J, Park C, Ahmed B, Young I, Frankis J, et al. Preparedness for use of the rapid result HIV self-test by gay men and other men who have sex with men (MSM): a mixed methods exploratory study among MSM and those involved in HIV prevention and care. HIV Med. 2017;18(4):245-55.

29. Godin G, Naccache H, Pelletier R. Seeking medical advice if HIV symptoms are suspected. Qualitative study of beliefs among HIV-negative gay men. Can Fam Physician. 2000;46:861-8.

30. Grandahl M, Larsson M, Herrmann B. 'To be on the safe side': a qualitative study regarding users' beliefs and experiences of internet-based self-sampling for Chlamydia trachomatis and Neisseria gonorrhoeae testing. BMJ Open. 2020;10(12):e041340.

31. Heijman T, Zuure F, Stolte I, Davidovich U. Motives and barriers to safer sex and regular STI testing among MSM soon after HIV diagnosis. BMC Infect Dis. 2017;17(1):194.

32. Heritage J, Jones M. A study of young peoples' attitudes to opportunistic Chlamydia testing in UK general practice. Reprod Health. 2008;5:11.

33. Hocking JS, Parker RM, Pavlin N, Fairley CK, Gunn JM. What needs to change to increase chlamydia screening in general practice in Australia? the views of general practitioners. BMC Public Health. 2008;8.

34. Hogan AH, Howell-Jones RS, Pottinger E, Wallace LM, McNulty CA. "...they should be offering it": a qualitative study to investigate young peoples' attitudes towards chlamydia screening in GP surgeries. BMC Public Health. 2010;10:616.

35. Jones LF, Ricketts E, Town K, Rugman C, Lecky D, Folkard K, et al. Chlamydia and HIV testing, contraception advice, and free condoms offered in general practice: a qualitative interview study of young adults' perceptions of this initiative. Br J Gen Pract. 2017;67(660):e490-e500.

36. Joore IK, van Roosmalen SL, van Bergen JE, van Dijk N. General practitioners' barriers and facilitators towards new provider-initiated HIV testing strategies: a qualitative study. Int J STD AIDS. 2017;28(5):459-66.

37. King EJ, Maman S, Dudina VI, Moracco KE, Bowling JM. Motivators and barriers to HIV testing among street-based female sex workers in St. Petersburg, Russia. Glob Public Health. 2017;12(7):876-91.

38. Krabbenborg N, Spijker R, Żakowicz AM, de Moraes M, Heijman T, de Coul EO. Community-based HIV testing in The Netherlands: experiences of lay providers and end users at a rapid HIV test checkpoint. AIDS Res Ther. 2021;18(1):34.

39. Lorch R, Hocking J, Guy R, Vaisey A, Wood A, Lewis D, et al. Practice nurse chlamydia testing in Australian general practice: a qualitative study of benefits, barriers and facilitators. BMC family practice. 2015;16(1):1‐10.

40. Lorimer K, Reid ME, Hart GJ. "It has to speak to people's everyday life...": qualitative study of men and women's willingness to participate in a non-medical approach to Chlamydia trachomatis screening. Sex Transm Infect. 2009;85(3):201-5.

41. Lorimer K, McDaid L. Young men's views toward the barriers and facilitators of Internet-based Chlamydia trachomatis screening: qualitative study. J Med Internet Res. 2013;15(12):e265.

42. Lorimer K, Martin S, McDaid LM. The views of general practitioners and practice nurses towards the barriers and facilitators of proactive, internet-based chlamydia screening for reaching young heterosexual men. BMC Fam Pract. 2014;15:127.

43. Malta M, Bastos FI, Strathdee SA, Cunnigham SD, Pilotto JH, Kerrigan D. Knowledge, perceived stigma, and care-seeking experiences for sexually transmitted infections: a qualitative study from the perspective of public clinic attendees in Rio de Janeiro, Brazil. BMC Public Health. 2007;7:18.

44. Manirankunda L, Loos J, Alou TA, Colebunders R, Nöstlinger C. "It's better not to know": perceived barriers to HIV voluntary counseling and testing among sub-Saharan African migrants in Belgium. AIDS Educ Prev. 2009;21(6):582-93.

45. Manirankunda L, Loos J, Debackaere P, Nöstlinger C. "It is not easy": challenges for provider-initiated HIV testing and counseling in Flanders, Belgium. AIDS Educ Prev. 2012;24(5):456-68.

46. Masaro CL, Johnson J, Chabot C, Shoveller J. STI service delivery in British Columbia, Canada; providers' views of their services to youth. BMC Health Serv Res. 2012;12:240.

47. McDonagh LK, Harwood H, Saunders JM, Cassell JA, Rait G. How to increase chlamydia testing in primary care: a qualitative exploration with young people and application of a meta-theoretical model. Sex Transm Infect. 2020;96(8):571-81.

48. McNulty CA, Freeman E, Bowen J, Shefras J, Fenton KA. Barriers to opportunistic chlamydia testing in primary care. Br J Gen Pract. 2004;54(504):508-14.

49. McNulty CA, Freeman E, Howell-Jones R, Hogan A, Randall S, Ford-Young W, et al. Overcoming the barriers to chlamydia screening in general practice--a qualitative study. Fam Pract. 2010;27(3):291-302.

50. Mills N, Daker-White G, Graham A, Campbell R. Population screening for Chlamydia trachomatis infection in the UK: a qualitative study of the experiences of those screened. Fam Pract. 2006;23(5):550-7.

51. Mitra D, Jacobsen MJ, O'Connor A, Pottie K, Tugwell P. Assessment of the decision support needs of women from HIV endemic countries regarding voluntary HIV testing in Canada. Patient Educ Couns. 2006;63(3):292-300.

52. Navaza B, Guionnet A, Navarro M, Estévez L, Pérez-Molina JA, López-Vélez R. Reluctance to do blood testing limits HIV diagnosis and appropriate health care of sub-Saharan African migrants living in Spain. AIDS Behav. 2012;16(1):30-5.

53. Normansell R, Drennan VM, Oakeshott P. Exploring access and attitudes to regular sexually transmitted infection screening: the views of young, multi-ethnic, inner-city, female students. Health Expect. 2016;19(2):322-30.

54. Oliver de Visser R, O'Neill N. Identifying and understanding barriers to sexually transmissible infection testing among young people. Sex Health. 2013;10(6):553-8.

55. Peters CMM, Dukers-Muijrers N, Evers YJ, Hoebe C. Barriers and facilitators to utilisation of public sexual healthcare services for male sex workers who have sex with men (MSW-MSM) in The Netherlands: a qualitative study. BMC Public Health. 2022;22(1):1398.

56. Prost A, Chopin M, McOwan A, Elam G, Dodds J, Macdonald N, et al. "There is such a thing as asking for trouble": taking rapid HIV testing to gay venues is fraught with challenges. Sex Transm Infect. 2007;83(3):185-8.

57. Prost A, Sseruma WS, Fakoya I, Arthur G, Taegtmeyer M, Njeri A, et al. HIV voluntary counselling and testing for African communities in London: learning from experiences in Kenya. Sex Transm Infect. 2007;83(7):547-51.

58. Rana J, Burchell AN, Wang S, Logie CH, Lisk R, Gesink D. Community perspectives on ideal bacterial STI testing services for gay, bisexual, and other men who have sex with men in Toronto, Canada: a qualitative study. BMC Health Serv Res. 2022;22(1):1194.

59. Reisen CA, Zea MC, Bianchi FT, Poppen PJ, del Río González AM, Romero RA, et al. HIV testing among MSM in Bogotá, Colombia: the role of structural and individual characteristics. AIDS Educ Prev. 2014;26(4):328-44.

60. Scheim AI, Travers R. Barriers and facilitators to HIV and sexually transmitted infections testing for gay, bisexual, and other transgender men who have sex with men. AIDS Care. 2017;29(8):990-5.

61. Seedat F, Hargreaves S, Friedland JS. Engaging new migrants in infectious disease screening: a qualitative semi-structured interview study of UK migrant community health-care leads. PLoS One. 2014;9(10):e108261.

62. Shangase P, Egbe CO. Barriers to accessing HIV services for Black African communities in Cambridgeshire, the United Kingdom. J Community Health. 2015;40(1):20-6.

63. Shoveller J, Johnson J, Rosenberg M, Greaves L, Patrick DM, Oliffe JL, et al. Youth's experiences with STI testing in four communities in British Columbia, Canada. Sex Transm Infect. 2009;85(5):397-401.

64. Somerset S, Evans C, Blake H. Accessing Voluntary HIV Testing in the Construction Industry: A Qualitative Analysis of Employee Interviews from the Test@Work Study. Int J Environ Res Public Health. 2021;18(8).

65. Thornton AC, Rayment M, Elam G, Atkins M, Jones R, Nardone A, et al. Exploring staff attitudes to routine HIV testing in non-traditional settings: a qualitative study in four healthcare facilities. Sex Transm Infect. 2012;88(8):601-6.

66. Uusküla A, Kangur K, McNutt LA. Barriers to effective STI screening in a post-Soviet society: results from a qualitative study. Sex Transm Infect. 2006;82(4):323-6.

67. Vaughan D, O'Connell E, Cormican M, Brugha R, Faherty C, Balfe M, et al. "Pee-in-a-Pot": acceptability and uptake of on-site chlamydia screening in a student population in the Republic of Ireland. BMC Infect Dis. 2010;10:325.

68. Wagg E, Hocking J, Tomnay J. What do young women living in regional and rural Victoria say about chlamydia testing? A qualitative study. Sex Health. 2020;17(2):160-6.

69. Wallace L, McNulty C, Hogan A, Bayley J. Exploring attitudes and practices of General Practice staff towards offers of opportunistic screening for chlamydia: a UK survey. Prim Health Care Res Dev. 2012;13(3):255-68.

70. Woodbridge MR, Dowell AC, Gray L. 'He said he had been out doing the traffic': general practitioner perceptions of sexually transmitted infection and HIV testing strategies for men. J Prim Health Care. 2015;7(1):50-6.
